# Eyebrow position in grammatical and emotional expressions in Kazakh-Russian Sign Language: A quantitative study

**DOI:** 10.1371/journal.pone.0233731

**Published:** 2020-06-02

**Authors:** Vadim Kimmelman, Alfarabi Imashev, Medet Mukushev, Anara Sandygulova

**Affiliations:** 1 Department of Linguistic, Literary, and Aesthetic Studies, University of Bergen, Bergen, Norway; 2 Department of Robotics and Mechatronics, School of Engineering and Digital Sciences, Nazarbayev University, Nur-Sultan, Kazakhstan; University of Massachusetts - Dartmouth, UNITED STATES

## Abstract

Facial expressions in sign languages are used to express grammatical functions, such as question marking, but can also be used to express emotions (either the signer’s own or in constructed action contexts). Emotions and grammatical functions can utilize the same articulators, and the combinations can be congruent or incongruent. For instance, surprise and polar questions can be marked by raised eyebrows, while anger is usually marked by lowered eyebrows. We investigated what happens when different emotions (neutral/surprise/anger) are combined with different sentence types (statement/polar question/wh-question) in Kazakh-Russian Sign Language (KRSL), replicating studies previously made for other sign languages. We asked 9 native signers (5 deaf, 4 hearing children of deaf adults) to sign 10 simple sentences in 9 conditions (3 emotions * 3 sentence types). We used OpenPose software to track eyebrow position in the video recordings. We found that emotions and sentence types influence eyebrow position in KRSL: eyebrows are raised for polar questions and surprise, and lowered for anger. There are also some interactions between the two factors, as well as some differences between hearing and deaf native signers, namely a smaller effect of polar questions for the deaf group, and a different interaction between emotions and wh-question marking in the two groups. We thus find evidence for the complex influences on non-manual behavior in signers of sign languages, and showcase a quantitative approach to this field.

## Introduction

Sign languages use facial expressions for a variety of reasons, including marking grammatical functions [1]. At the same time, signers can also express emotions through facial expressions, either their own, or, more importantly, of someone else in the context of constructed action or speech, when the signer quotes someone [2]. Since facial expressions can be used to express different functions, sometimes a clash between the functions can occur. For instance, in many sign languages, polar questions are marked with raised eyebrows [3]. At the same time, angry facial expression commonly includes lowered (furrowed) eyebrows [4]. What happens if the signer asks an angry polar question: will the eyebrows be raised, or lowered, both, or neither?

Researchers have investigated this question using data from American Sign Language (ASL) [5] and Sign Language of the Netherlands (NGT) [6] with some non-trivial findings, see also below. In this paper, we contribute to this question by investigating interactions of emotional and grammatical factors on eyebrow position in yet another sign language, Kazakh-Russian Sign Language (KRSL), which has not been investigated previously in this respect. In addition, we use the OpenPose software [7–10] in order to analyze eyebrow positions quantitatively and with high precision, which was previously not possible due to the lack of appropriate technology. Thus the study can also be considered a showcase of the future of quantitative research on non-manual markers in sign languages.

### Non-manual markers in sign languages

It is a common mistake to think that sign languages are languages produced by hands. In fact, sign language users employ non-manual articulations (body leans and turns, head leans, turns, shakes, nods, mouth movements, eyelid movements, nose wrinkling, eyebrow movements, etc.) for a variety of linguistic functions, see [1] for an overview. For instance, mouth movements can be used for lexical disambiguation, or for expressing adverbial meanings; body movements can be used to express contrast and mark constructed action or speech; head movements are commonly used for negation and affirmation. The focus of this paper is eyebrow movements, which can also be used for a variety of functions.

More specifically, eyebrow movements are commonly used to mark sentence type (statement vs. question) [3], to mark topics and foci [11, 12], and to mark subordinate clauses of various types [13]. It is important to realize that eyebrow movements are usually combined with other non-manual markers in these functions. For instance, topic marking in some sign languages involves not only raised eyebrows, but also backward head leans, and eyes wide open [14].

In many sign languages, statements are characterized by neutral eyebrow positions (unless there are other factors that cause eyebrow movement, such as subordinate status of the clause, etc.). Polar questions are most commonly marked by raised eyebrows (as well as by some type of head movement) [3], but see also [15]. The marking of wh-questions is more mixed: in some languages, eyebrow lowering is employed [16], while in others, eyebrow raising [17], or both can be used [18]. For KRSL, no previous research has been done, while in RSL, some preliminary findings indicated the use of eyebrow raise in polar questions, and both eyebrow raise and lowering in wh-questions.

Another important phenomenon that is marked non-manually in sign languages is the so-called constructed actions/speech [2, 19, 20]. When the signer wants to convey someone’s speech or actions, they can take on the role of the other person (or animal) so that the signer’s signs, gestures, and facial expressions would be attributed to this other person. This is sometimes achieved by turning the body and the head, and shifting eye gaze to the side (the so-called *role shift*), but these non-manual markers are not obligatory, at least not in RSL [20].

Eyebrow movements per se are not involved in role shift. However, if the signer is quoting someone experiencing emotions, they might also represent an emotional facial expression of the quoted person. So, for instance, if the quoted person is angry, the signer might choose an angry facial expression. Note that this use of emotional facial expressions is thus performative and expressive, and not a manifestation of real emotions of the signer producing the expression.

### Interaction between grammar and emotions

Facial expressions sometimes represent emotions. Note that there is a lot of debate which emotions are expressed by facial movements, how exactly this happens, and whether facial expressions are reliable and specific markers of emotions (see [21] for a recent critical overview), and the issue of the existence of basic universal emotions is also contested [22]. Irrespective of whether people in fact express their own emotions reliably with specific facial expressions, there are clear cultural conventions associating some emotions with certain facial expressions. These conventions are encoded among others in movies, paintings, animated movies, emoji symbols, etc.

Sign language users can use the conventions around emotional facial expressions to represent emotions of the people they quote. In addition, it is also very likely that signers’ actual emotions affect their facial expressions, but this is left out of consideration in this study.

Some of the emotions are typically associated with eyebrow movements: surprise with raised eyebrows (both inner and outer parts), and anger with furrowed eyebrows (lowering the inner part of the eyebrow, and moving eyebrows closer together) [4]. Given that eyebrow movement is also controlled by sentence type, a clash might arise if a sentence is uttered in a particular sentence type, and with a particular emotion. A small number of studies have investigated what happens in such situations.

De Vos et al. [6] investigated effects of emotions (neutral, anger, surprise) on eyebrow position in polar questions and wh-questions in Sign Language of the Netherlands (NGT). They elicited declaratives, sentences with marked topics, polar questions, and wh-questions in combination with three emotions from two native NGT signers, but for the analysis they focused on two sentence types (polar questions and wh-questions) with three emotions. The eyebrow position was manually coded using the Facial Action Coding System (FACS) [23] for action type and action intensity. Previous research had identified AU 1 + AU 2 (inner and outer brow raise) for polar questions and for expressions of surprise, and AU 4 (inner brow lowering) for wh-questions and anger. The authors were testing the “Affect over Grammar” hypothesis: they expected that affective expressions will be more important than linguistic ones, so, in angry polar questions, they expected to see AU 4, and, in surprised wh-questions they expected to see AU 1 + AU 2. Their findings were not in agreement with their hypothesis; instead, they found that brow lowering (whether related to anger or marking wh-questions) was preserved in competition with eyebrow raise, which they explained as AU 4 being phonetically stronger. In the context of congruent emotion-sentence type combinations (surprised polar questions, angry wh-questions), they found phonetic enhancement, that is, stronger eyebrow raise or lowering than with the neutral emotion.

Weast [5] investigated a similar research question, but for American Sign Language (ASL). She considered three sentence types (statement, polar question, wh-question) and five emotions (neutral, happy, sad, angry, surprised). She elicited 270 sentences from 6 native ASL signers (three different sentences with each combination of emotion and sentence type per person). In contrast to De Vos et al., she did not use FACS, but instead used the Screen Calipers (http://www.iconico.com/caliper/) digital tool to measure the distance in pixels between the middle position of the eyebrow and the eye in pixels. She found an intriguing pattern. First, emotions reliably determined eyebrow position in the predicted direction. Second, while in neutral sentences polar questions, statements, and wh-questions are reliably distinguished by eyebrow height average per sentence, in emotionally marked sentences the differences become smaller and not significant. Thus, in ASL, the “Affect over Grammar” hypothesis seems to find confirmation. However, Weast also argued that when looking at temporal organization of eyebrow position throughout the sentence (the dynamic contours), sentence types are in fact marked distinctly, even in the presence of emotion. Thus, when sentence type marking is combined with emotions, the type of dynamic contour distinguishes sentence types from each other, not the overall average eyebrow position.

Note that the issue of interaction between emotion and grammar in prosody is not limited to sign languages. Spoken languages use prosody (e.g. pitch or fundamental frequency (f0) modification and syllable duration) to convey both emotions and linguistic information, such as sentence type, information structure, and others. Because prosodic features of speech are affected by both grammar and emotion, they can interact. Pell [24] studied interaction of emotion, sentence type, and focus placement in Canadian English using a design similar to the studies on sign languages described above. He found main effects of emotions, focus, and sentence type on f0 and duration, but, most importantly, some non-trivial interactions. For instance, emotions were not reliably distinguished by f0 on words that were focused in questions, so f0 was primarily used to mark focus and sentence type, not emotion.

Given the limited number of studies in both signed and spoken languages, no clear conclusion emerges on the interaction between emotion and grammar in vocal and non-manual prosody. However, all studies indicate that this issue is worth pursuing, as non-trivial interactions are usually discovered.

### Kazakh-Russian Sign Language

In this study, we investigate Kazakh-Russian Sign Language (KRSL), that is, the sign language used in the Republic of Kazakhstan. KRSL is closely related to Russian Sign Language (RSL) and some other sign languages of the ex-Soviet Union. Kazakhstan used to be under the influence of the Russian Empire and later a part of the Soviet Union (until 1991), whose centralized language policy also led to the spread of RSL in the Soviet republics.

While no official research comparing KRSL with RSL exists, our observations based on our experience researching both languages is that they show a substantial lexical overlap, and are completely mutually intelligible.

KRSL has not been previously investigated linguistically (unlike RSL, for which a number of studies have been published, see [25] and references therein). We would like to point out that while RSL and KRSL are very similar lexically, we do not know if the same applies to the grammars of the two languages, including how they non-manually mark questions and represent emotions in constructed action contexts. We therefore do not claim that our findings for KRSL generalize to RSL.

According to some estimates, there are 150000 to 200000 deaf people living in Kazakhstan (see here and here). Russian Sign Language received the status of Official State Language in 2013 (see here), whereas KRSL still does not have enough legal normative documents to become an official language (see here). There are more than ten big public associations for deaf, deaf-blind, and hard of hearing people and their families, the largest and oldest one is the Kazakh Deaf Society. It was established in 1937 and has branches in almost all large cities of Kazakhstan. Also, there are special and boarding schools for children with hearing issues in each regional center supported by the government. Kazakhstan’s government provides 60 free hours per year of sign language interpreter service support for each deaf person, which could be spent on medical, legal, or other communication needs. In addition to disability support pension, the government also supplies personal computers and mobile phones to deaf individual every five years.

### The current study

In the current study, we investigate how emotional expressions and sentence types influence eyebrow position in KRSL and how they interact. We decided to focus on only three emotional expressions (neutral, anger, and surprise) and on three sentence types (statements, polar questions, wh-questions), as these emotions and sentence types have been studied before, and we had a clear expectation of their individual influence on eyebrow position.

The three emotions and sentence types form 9 cases, in two of which emotions and grammar are congruent (that is, they are expected to cause eyebrow movement in the same direction), in two—incongruent (expected to cause eyebrow movement in the opposite direction), and in five cases either an emotional or grammatical type is not expected to affect eyebrow position. This is represented in Table 1, where each cell represents an expected eyebrow movement from both sources.

**Table 1:** Expected eyebrow movement in combinations of emotions and sentence types.

	Surprise	Neutral	Anger
**Polar question**	up + up	up	up + down
**Statement**	up		down
**Wh-question**	down + up	down	down + down

Based on previous research, we might expect different things to happen in the congruent and non-congruent combinations. The “Affect over Grammar” hypothesis would predict that the emotional expression would win in the non-congruent cases. If KRSL behaves similarly to NGT, we might find eyebrow lowering overriding eyebrow raise in the contexts where they co-occur. On the other hand, it might be the case that KRSL marks both emotions and sentence types in a clear and distinct way, with no interaction between the two. Given the absence of previous research for KRSL, we cannot estimate which of the hypotheses is more likely in advance. We are thus interested in how emotions, sentence types, and their interaction affect eyebrow position in KRSL in this exploratory research.

## Materials and methods

### Design of the study

Based on prior work and our knowledge of KRSL, we selected three emotions (neutral, anger, surprise) and three sentence types (statement, polar question, wh-question) to study their influence on eyebrow position. The study was approved by the Ethics Board of Nazarbayev University, Nur-Sultan, Kazakhstan, where the data was collected.

We created 10 simple sentences consisting of a subject and an intransitive verb (see S1 Table for the full list). These sentences in the statement form and the polar question form contained two signs each in the same order (1a,b). In wh-questions, a single question sign is added in each case (in the first position in the sentence, which was the correct position according to our native signer consultants) (1c).

1.
  - GIRL FALL.
    ‘The girl fell.’
  - GIRL FALL?
    ‘Did the girl fall.’
  - WHERE GIRL FALL?
    ‘Where did the girl fall.’

The sentence were originally created in written Russian, and translated to KRSL by a native hearing KRSL signer with neutral emotion; the translations were recorded to be used as stimuli.

The participants (see below) were asked to watch each sentence in each form, and then sign it with three different emotions. See S1 Video for a recording of the nine possible combinations for one sentence produced by one of the signers. The individual in this recording has given written informed consent (as outlined in PLOS consent form) to publish the potentially identifying information (the recording showing their face).

We did not use any fillers and did not try to mislead the signers as explicit instructions were required to make them produce constructed emotional expressions. At the same time, we did not disclose that we were specifically interested in interactions between emotions and grammar on non-manual marking, although the participants were likely to have guessed it.

In order to avoid fatigue, participants took frequent breaks, including obligatory breaks between showing stimuli with different emotions. In addition, we used two different orders of stimuli presentation with different participants to counterbalance for possible fatigue effects.

After finishing the task, the participants had the opportunity to ask questions and/or provide feedback. Some of the signers provided feedback, mainly stating that the study was interesting and allowed them to reflect on their use of facial expressions.

### Participants

Nine native signers participated in the study. We define native signers as signers who grew up with at least one deaf signing parent and learned KRSL from the parent in childhood. Four of the signers are hearing children of deaf adults (CODA’s) currently working as KRSL interpreters. Five of the signers are deaf. The relevant information about the signers is provided in Table 2.

**Table 2:** Demographic information of the participants.

ID	Gender	Age	Deaf?	Current Region (Place of Origin)
S1	M	37	Yes	Nur-Sultan (Almaty)
S2	F	23	Yes	Nur-Sultan (Karagandy)
S3	F	45	Yes	Nur-Sultan (Atbasar)
S4	F	28	Yes	Nur-Sultan (Atbasar)
S5	F	35	Yes	Nur-Sultan (Taldyqorgan)
S6	F	34	No	Nur-Sultan
S7	F	35	No	Nur-Sultan
S8	M	40	No	Nur-Sultan (Almaty)
S9	F	27	No	Nur-Sultan (Aqmola region, Zhibek Zholy)

In addition to doing the experimental task, the participants filled in a short demographic questionnaire, and signed an Informed Consent Form, acknowledging their participation in the experiment, and giving us permission to use the video recordings for research and publication. The Informed Consent Form was provided in written form to the hearing participants, and in written and signed form to the deaf participants to ensure accessibility.

The fact that we included both hearing and deaf signers was initially based on availability of native signers. However, the additional question of whether hearing and deaf native signers are different from each other can thus also be investigated in this study. While both hearing and native signers in our sample are native and learned KRSL from their parents, their exposure to both KRSL, and especially to spoken languages might be different, which could also be reflected in their language use.

### Data collection

We created instructions for the participants in Russian, to be used with the hearing signers. An experienced interpreter (who is also a native KRSL signer) translated these instructions into KRSL, and the video recording of the instructions was shown to the deaf participants.

The instructions were as follows. “You will be asked to sign sentences in KRSL: statements, polar questions, and wh-questions. You will need to sign each sentence with three different emotions: neutral, surprised, and angry. Each sentence with each emotion has to be signed 10 times.” The hearing signers were asked to translate each sentence into KRSL from the written list. The deaf signers were shown the recorded KRSL versions of each sentence signed with neutral emotion by the interpreter.

Note that each sentence-emotion combination was asked to be signed 10 times because this data set was collected not only for the purposes of the research reported here, but also to develop a neural network model for automatic sentence type and emotion recognition. For this project, only one middle instance of a sentence-emotion combination by each signer was included.

For the hearing participants, there were two people in the room during each filming session: the participant and a research assistant to control the filming process and answer any questions. For the deaf participants, there were three people in the room: the participant, the research assistant to control the filming process, and an interpreter in the case the signer would have additional questions.

We used a set up with a green solid uniform background, a Logitech C922 camera (HD 1080 recording quality), and office-like illumination. The filming of each signer took 2.5-3 hours on average.

### Validation

In order to test whether the participants in our study indeed performed the intended emotions, we conducted a small validation experiment. We randomly selected 81 videos (9 from each signer) to represent different emotions and sentence types. We then showed these videos to five more deaf signers, who did not participate in the main data collection. The signers were 3 males, 2 females, mean age = 24. The participants of the validation task were asked to identify the emotion of the signer in the video by selecting between four options: neutral, surprised, angry, other. The order of videos in the experiment was randomized, and two different orders were created.

The full analysis is included in S2 Appendix. We conducted analysis of the data in R (version 3.6.1) [26] using R Studio (version 1.2.5019) [27]. The overall results are as follows. Overall, we find that emotions were identified correctly (that is, got the intended label) in 61% of cases (which is higher than expected chance agreement of 33% or 25% if we take into account the *other* category). Thus the correct identification in our task is much higher than in De Vos et al.’s study [6]. Neutral emotion is identified slightly less accurately than anger or surprise.

Using Cohen’s kappa to account for chance agreement, we calculated agreement between intended labels and each of the participant’s judgments, using the *cohen.kappa* function from *psych* package, version 1.8.12 [28]. The results range from 0.39 to 0.56 (all significant) which indicates fair to moderate agreement [29]. Agreement between the five participants corrected by chance estimated by Light’s kappa (calculated using the *kappam.light* function from the *irr* package, version 0.84.1, https://cran.r-project.org/package=irr) is 0.398, p = 0.00564. Thus we can conclude that the emotions were indeed recognized significantly higher than would be expected by chance.

In the literature on emotion recognition, a different measure of agreement corrected by chance is often used [30], which is calculated using the following formula: proportion correct—(1/number of choices))/ (1—(1/number of choices)). In order to be able to compare our results to other studies, we also used this formula. If we take the number of categories to be 3, we get the proportion of 0.42, and if we consider the number of categories to be 4, we get the proportion of 0.48. This is somewhat lower than what is reported for emotion identification using video stimuli for spoken languages [30], which is 0.64 on average.

The somewhat lower than expected accuracy of emotion identification in our data might in fact be caused by interactions between emotional and grammatical marking which we describe in the next section. This is confirmed by looking at the raw proportions of correct answers for statements (73%) vs. polar and wh-questions (58% and 56%). However, it is clear that the intended emotions are still identifiable at a higher than chance level even in the presence of grammatical marking competing for the use of the same non-manual articulators.

### Video analysis

We detected and extracted keypoints of face, hands and pose from videos using OpenPose Human Pose Estimation library (version 1.5.0-binaries-win64-gpu) [7–10]. OpenPose Demo is an executable version of the library that can easily process videos and save results. It was run on Windows 10 and Nvidia GeForce GTX 1080 GPU card with 8GB of memory. Additional parameters *–net_resolution 320x240* and *–number_people_max 1* were used to increase processing speed, as all videos had only one person, and to make fixed net resolution.

Videos were processed frame by frame (at 30 fps) and results were saved in a JSON format file for each frame. Extracted data had x,y-coordinates and confidence score for 70 keypoints of face, including 5 keypoints for each eyebrow, 21 keypoints for each hand, and 24 keypoints for pose (see the OpenPose documentation for keypoint locations here). Finally, all extracted data was combined in one CSV format file in Python (version 3.6) using its standard libraries.

### Statistical analysis

We conducted analysis of the data in R (version 3.6.3) [26] using R Studio (version 1.0.143) [27]. The RMarkdown script and all the data files are available in S1 Appendix and can be used to replicate the analysis.

The exported keypoint data from OpenPose contains x and y coordinates for 5 keypoints on each of the eyebrows, as well as confidence levels (0 to 1) provided by OpenPose and showing how confident the software is of each measurement. We removed all the keypoints with confidence level below 0.7 from the data set.

We are interested in vertical eyebrow movement; however, using raw y-coordinates is not appropriate because they simply indicate the distance from the eyebrow to the frame boundary, which is affected by the signers height, the camera position, the body position, body and head movements, etc. We therefore use a relative measure for eyebrow position, namely the distance between the the eyebrow keypoint and the keypoint the top of the nose (keypoint 27) based on both x- and y-coordinates.

We also restricted the data by removing the first and last 20% of each video file. The reason for doing it was that signers were raising and lowering their hands in the beginning and ending parts of the videos (as we determined quantitatively based on the hands’ keypoint data, see the details in S1 Appendix). These preparatory and retractory activities do not belong to the sentence proper, and the non-manual markers might not occur in these periods.

For the statistical modeling we decided not to model each keypoint separately because it is clear that their movements are not independent from each other. Instead, based on the facial anatomy, we only investigate two dependent variables: internal eyebrow position and external eyebrow position, as these parts of the eyebrows can move partially independently (that is, they are raised separately by AU 1 and AU 2 [23]. The internal eyebrow position variable was created by averaging between heights of keypoints 21 and 22 (for the two eyebrows), and the external eyebrow variable was created by averaging between heights of keypoints 18 and 25 (for the two eyebrows). We average the heights for two eyebrows because the eyebrows are not always fully symmetric. In the statistical analysis (S1 Appendix) we also investigate this asymmetry and show that it is related to some of the factors under investigation. However, we do not discuss this issue further in this paper.

For this first study we decided to focus on average eyebrow position for each video and not analyse the dynamic contour within each video, as the latter would require advanced quantitative techniques that have not been developed yet for such purposes to the best of our knowledge. Thus, we averaged the eyebrow position in each video file. We ended up with 805 measurements for each of the two variables. The total number of sentences should be 810, but 5 sentences contained no data points after removing the first and last 20% of the duration, and the data points with confidence level below 0.7.

Given the design of the study, a mixed effects multivariate linear regression model is suitable. The outcome variables are internal and external relative eyebrow positions (in pixels). The fixed predictor variables are sentence type (categorical, three levels), emotion (categorical, three levels), group (categorical, deaf vs. hearing), and all the interactions between the three predictors. Finally, the random variables are participant (with random slopes for sentence type, emotions, and their interactions), and sentence (with random slopes for emotions, group, and their interactions) (1).

$$\begin{array}{c} \begin{array}{c} internal/external∼emotion*sentence.type*group\\ +(emotion*group|sentence)+(emotion*sentence.type|speaker.id) \end{array} \end{array}$$ (1)

A common tool to build mixed effects linear regression in R is the *lme4* package [31]. However, it has been found that with a small number of levels for random effects, the models often result in a singular fit [32]. As a solution, it is possible to impose a covariance prior so that a singular estimate of the covariance matrix of the random coefficients becomes impossible [32]. This can be done with the *blme* package [33], which we used for this study (version 1.0-4). Significance of the contribution of the different factors was determined using the *Anova* function from the *car* package, version 3.0-7 [34].

## Results

**Fig 1:**
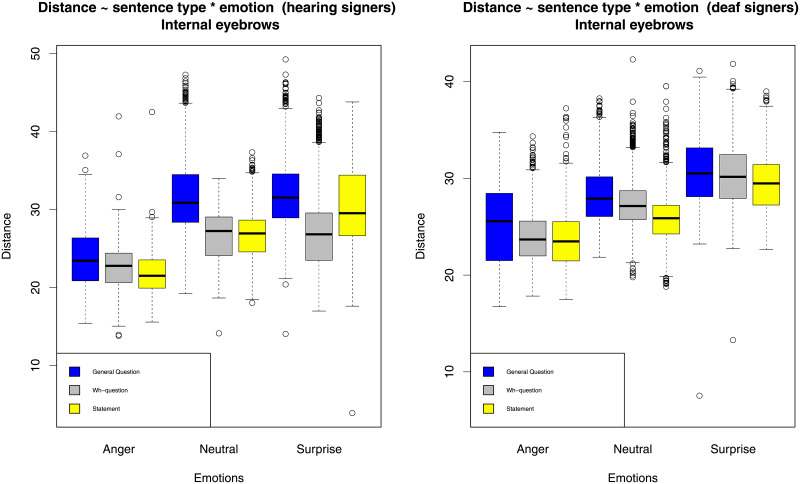
Internal eyebrow height.

**Fig 2:**
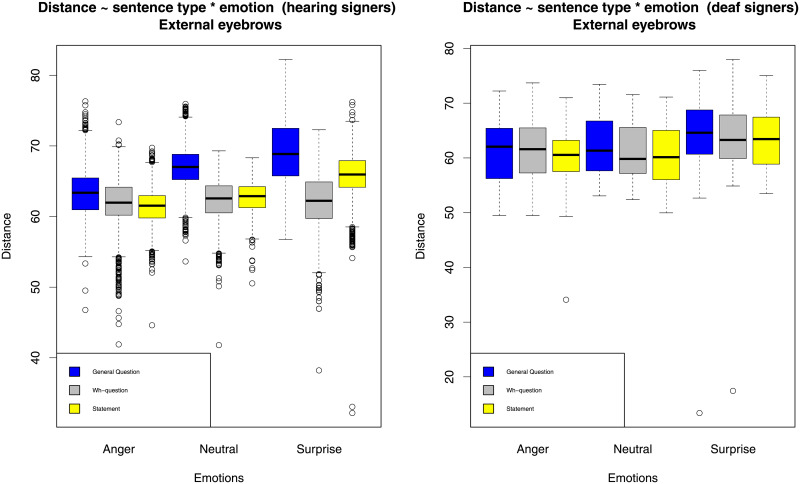
External eyebrow height.

As the first step, we graphically explored the data by plotting the average eyebrow distance from the nose (separately for internal and external eyebrows) for the two groups of signers and across different conditions. The results are represented in Figs 1 and 2.

From these figures, several observations can be made. First, emotions clearly determine eyebrow position in the expected directions: anger lowers eyebrows, while surprise raises eyebrows. Second, sentence types also determine eyebrow position (polar questions raise eyebrows in comparison to statements), but to a lesser extent, and the difference between statements and wh-questions seems to be small. Third, there seem to be some interactions between emotions and sentence types. Fourth, we observe some differences between hearing and deaf signers. In order to numerically assess these various effects, we applied statistical modeling. The full results of the statistical analysis can be seen in the output file (S1 Appendix).

We find that emotions significantly influence both the internal eyebrow position (*χ*^2^ = 68.9, *df* = 2, *p* < 0.001), and the external eyebrow position (*χ*^2^ = 32.35, *df* = 2, *p* < 0.001), in the expected direction. For internal eyebrow position, the eyebrows are higher for neutral than angry emotions by estimated 3.7 pixels (*se* = 0.8, *t* = 4.5), and the average between neutral and angry is lower than surprise by estimated 4 pixels (*se* = 0.63, *t* = 6.6). For external eyebrow position, the eyebrows are higher for neutral than angry emotions by estimated 0.45 pixels with high variation (*se* = 0.8, *t* = 0.55), and the average between neutral and angry is lower than surprise by estimated 2.5 pixels (*se* = 0.48, *t* = 5.2). The effect of anger on the external eyebrow position thus is small and likely due to chance, while for surprise both internal and external eyebrows are involved. This confirms the well-known observation that anger is expressed by AU 4 (inner eyebrow lowering), while surprise is expressed by AU 1 + AU 2 (inner and outer eyebrow raise).

We also find that sentence type significantly influences both internal (*χ*^2^ = 78.3, *df* = 2, *p* < 0.001) and external (*χ*^2^ = 33.9, *df* = 2, *p* < 0.001) eyebrow positions. However, when we focus on the individual contributions of the three sentence types, it is clear that a large and significant difference is only found for polar questions, which cause both internal and external eyebrows to raise, while wh-questions are very similar to statements with respect to eyebrow positions. For internal eyebrow position, the eyebrows are higher (not lower as expected) for wh-questions than statements by estimated 0.3 pixels (*se* = 0.38, *t* = 0.8), and the average between statement and wh-question is lower than polar questions by estimated 2.3 pixels (*se* = 0.28, *t* = 8.2). For external eyebrow position, the eyebrows are very slightly higher for wh-questions than statements by estimated 0.09 pixels (*se* = 0.39, *t* = 0.22), and the average between statement and wh-question is lower than polar questions by estimated 2.5 pixels (*se* = 0.4, *t* = 6). We have to conclude that, in KRSL, wh-questions are not consistently marked by lowered eyebrows.

The main effect of group is not significant for either internal or external eyebrow positions. This is expected, as there is no reason to assume that eyebrows are on average higher or lower for deaf vs. hearing individuals.

Turning on to the interactions, we find significant interactions for both internal and external eyebrow positions, but they are different for the two cases.

For internal eyebrow positions, the only significant interaction is between sentence type and group (*χ*^2^ = 24, *df* = 2, *p* < 0.001). Looking at the results of the regression model, we can see that this is due to the difference in marking of polar questions: the raise observed for the deaf signers is smaller than for the hearing signers (estimate is 1.8 pixels, *se* = 0.6, *t* = 3.1). This means that while both groups raise the internal parts of the eyebrows for polar questions, the hearing signers in our sample produce a higher eyebrow raise. Counter to our expectations, we did not find a significant interaction between emotions and sentence types.

For external eyebrow position, there is a significant interaction between sentence type and group (*χ*^2^ = 8.9, *df* = 2, *p* = 0.012), a significant interaction between sentence type and emotion (*χ*^2^ = 11.3, *df* = 4, *p* = 0.024), and a significant three-way interaction, (*χ*^2^ = 10.5, *df* = 4, *p* = 0.033). Looking at the results of regression model, we see that the interaction between sentence type and group is due to the contribution of both polar questions and wh-questions. The difference between polar questions and the average of the other two sentence types is smaller for deaf signers than for hearing signers (estimate 2.3 pixels *se* = 0.82, *t* = 2.8). Looking back at the figures, we can see that deaf signers employ external eyebrow raise for polar questions to a lesser degree than hearing signers. In addition, the main effect of wh-questions is lower in the deaf group (estimate 1.8 pixels *se* = 0.8, *t* = 2.3) because the deaf signers do lower eyebrows somewhat in wh-questions, while the hearing signers do not.

The interaction between sentence type and emotion for external eyebrow positions can be traced back to the effect of surprise on wh-questions. The eyebrow raise difference between wh-questions and statements is counteracted by the presence of surprise (estimate 2 pixels, *se* = 0.56, *t* = 3.5). This is due to the fact that the hearing signers mark surprised statements with higher eyebrow raise than surprised wh-questions, while for neutral statements and wh-questions the pattern is reversed. This is also the reason for the significant three-way interaction driven by the difference in wh-questions with surprise in the two groups (estimate 3.3 pixels, *se* = 1.1, *t* = 3).

To sum up, we find that KRSL signers use eyebrows to perform emotional facial expressions in the expected manner. In addition, they use eyebrow raise to mark polar questions. At the same time, wh-questions are not consistently marked with eyebrow lowering. We found interactions between grammatical and emotional marking, but only for external eyebrows: wh-questions behave unexpectedly in combination with surprise. Finally and unexpectedly, we found interactions between the group (deaf vs. hearing signers) and the marking of sentence types and emotions. Deaf signers seem to mark polar questions to a lesser extent than hearing signers, and hearing signers show a different pattern in marking wh-questions in combination with surprise.

## Discussion

We found some expected and some unexpected patterns in eyebrow movement in KRSL signers.

First, when KRSL signers are asked to produce emotional speech, they indeed produce facial expressions that are expected based on existing research on other cultures [30]. Specifically, inner and outer eyebrow raise is used to represent surprise, and inner eyebrow lowering is used to represent anger. This finding is not surprising, but it does make a small contribution to the large body of evidence that there are at least culturally established links between specific emotions and facial expressions, and the facial expressions conventionally associated with emotions also occur in sign language users.

Second, we found that KRSL signers use eyebrow position to mark sentence type. In agreement with our expectations, and with observations for many other sign languages [18], KRSL signers raise eyebrows in polar questions. However, in wh-questions, we did not observe eyebrow lowering. Instead, eyebrow position on average does not distinguish wh-questions from statements. This is not entirely unexpected. Several sign languages have been reported to have a mixed eyebrow movement pattern in wh-questions, including some recent findings for languages that have been previously claimed to consistently mark these questions [15]. Note that we are not claiming that wh-questions in KRSL are not non-manually marked, as we did not analyze other non-manual features. In fact, when we informally looked at the dataset, it appeared that the main non-manual marker of wh-questions in KRSL is chin raise (or backward head lean) on the wh-sign.

The core question that we wanted to investigate was potential interactions between emotions and sentence type marking, especially in non-congruent contexts. Our findings indicate that emotional marking has an effect on grammatical marking for outer (but not inner) eyebrow position. Specifically, the movement pattern in wh-questions is reversed in surprise contexts. The fact that there is a significant interaction means that eyebrow position cannot be determined by simply adding (or subtracting) the effects of emotions and sentence types. We do not find direct support for the “Affect over Grammar” hypothesis [6] because we do not observe that emotional expressions completely override grammatical marking, but we observe interactions (also with the deaf vs. hearing signers). In addition, given the statistical analysis, it is impossible to say whether it is emotions that are inhibiting/reversing grammatical marking, or that different grammatical marking can affect expression of emotions.

In addition, we accidentally discovered a difference between the hearing and deaf signers in our sample. It turns out that the deaf signers marked questions and emotions with smaller eyebrow position changes than the hearing signers (which is especially visible for external eyebrows). Prior to the experiment we did not expect to find any such differences. At first sight, it seems mysterious why hearing native signers would use larger eyebrow movements to mark sentence types than deaf native signers. However, we do have a tentative explanation. The hearing signers in our sample are all KRSL interpreters. In their professional work, they are expected to sign in a very clear and precise manner, and this also concerns their facial expressions. Therefore, in our task, they produced facial expressions that were exaggerated in comparison to the more natural and less pronounced facial expressions by the deaf signers. At this stage this is only a post-hoc explanation that we did not test, but it can be tested in future. It is important to note that despite the differences in amplitude of eyebrow movement between the groups, the same direction of movement was observed for both emotional and grammatical purposes in the two groups, so the two groups of signers do not have distinct systems of grammatical or emotional eyebrow movements.

The PLOS ONE reviewers have suggested two alternative explanations for the observed difference between the hearing and deaf signers, both related to the elicitation procedure. The hearing signers were presented with written stimuli, while the deaf signers were presented with stimuli in KRSL. It is not clear, however, why written stimuli would produce a more pronounced non-manual component. In addition, the presence of the hearing researcher during the recording sessions could have led the deaf participants to mitigate their non-manual component. We cannot either confirm or reject these explanations, but we can conclude that future studies should take these possible factors into account when designing the elicitation procedure.

## Conclusions

In this paper we reported the results of first ever quantitative investigation of eyebrow position affected by grammar and emotions in KRSL. We collected a dataset of 10 sentences signed in three different sentence types (statement, polar question, wh-question) and with three different emotions (neutral, anger, surprise) signed by 9 native signers. We aimed at replicating findings for other sign languages (ASL [5], NGT [6]) but with larger samples, and with higher precision and reliability using a new automatic annotation of eyebrow position with the help of OpenPose.

We found that eyebrow position is indeed affected by emotions (eyebrows are raised for surprise, and internal parts of eyebrows are lowered for anger), and by sentence type (polar questions are marked by eyebrow raise). We also found an interaction between emotions and sentence type, and also some differences between the hearing and deaf signers in our sample, which we attribute to professional occupation of the hearing signers.

A practical purpose of this paper was to demonstrate the advantages of using novel computer tools to analyze sign language data. With tools such as OpenPose, it is now possible to automatically and precisely track articulators in 2D video recordings, which in turn makes it possible to analyze large data sets of sign language data.

It is clear that our study has some limitation which should be addressed in future research. First, we used a relatively small sample of signers (albeit a larger one than in similar previous studies), and we unintentionally found differences between hearing and deaf native signers. In order to find our whether our proposed explanation for the difference is valid, a further study is necessary where the professional occupation of the signers is a factor that is controlled for.

Another obvious limitation is that we only looked at average eyebrow position in each sentence, without taking into account dynamic changes. Weast [5] found that, in ASL, sentence type marking disappears in the presence of emotional marking, but only when looking at average position, while the dynamic prosodic contours distinguish sentence types also in the presence of emotions. However, Weast’s study of dynamic prosodic contours was basically qualitative, as not statistical analysis was provided. Our data set can be used for quantitative analysis of dynamic contours. However, this requires a much more advanced analysis than the one attempted in this paper. For now we must state that the findings made in this study are only about sentence-average eyebrow positions, and that a future study is likely to discover much more intricate patterns.

Finally, in this study, we only looked at eyebrow position, while it is clear that both emotional expressions [4] and grammatical sentence-type non-manual markers [1] also involve other articulators, such as eye aperture, head turns and tilts, chin position, and body movement and position. We hope that in future with the development of automatic methods it will be possible to study grammatical non-manuals and their interactions with emotional expressions in a more holistic way.

## Supporting information

S1 Appendix Statistical analysis. This Appendix contains the statistical analysis (in RMarkdown format and exported to html) and the full data tables used in the analysis.
(ZIP)

S2 Appendix Validation analysis. This Appendix contains the statistical analysis (in RMarkdown format and exported to html) of the validation experiment and the full data table used in the analysis.
(ZIP)

S1 Table S1 Table contains the sentences used in this study. (PDF)

S1 Video Video illustrating target utterances. This video contains the same sentence (‘The girl fell down’) signed as three different sentence types and with three different emotions (9 total combinations) by one of the participants of the study.
(MP4)

S2 Video Video illustrating OpenPose results. This is the same video as in S1 Video. (one sentence with three sentence types and three emotions) with OpenPose keypoints overlaid, demonstrating the quality of OpenPose’s tracking of the body, hands, and facial features.
(AVI)
