# Supplementary material for: Eyebrow position in grammatical and emotional expressions in Kazakh-Russian Sign Language: A quantitative study
Source: PLoS One. 2020 Jun 2;15(6):e0233731. doi: 10.1371/journal.pone.0233731 (PMC7266324; doi:10.1371/journal.pone.0233731)
Supplement: S1 Appendix — This Appendix contains the statistical analysis (in RMarkdown format and exported to html) and the full data tables used in the analysis. (ZIP) [file pone.0233731.s001.zip › analysis-clean-revised.html]

Eyebrow analysis


Code 

- Show All Code
- Hide All Code

# Eyebrow analysis

#### Vadim Kimmelman

#### 04/2020

---

# Analysis

This is the code used for the statistical analysis of the keypoint data exported from OpenPose for videos recorded to study the interaction of grammatical marking and emotions on eyebrow position in Kazakh Russian Sign Language (KRSL).

The dependent variables that we are interested in are positions of different parts of the eyebrows. The predictor variables are grammatical sentence type (statement, polar question, wh-question) and emotions (neutral, anger, surprise). Due to the experimental design, we treat and deaf and hearing groups of signers as another predictor, and we also have to treat sentence and signer as random factors.

The following data files are required:

- keypoints2.csv (data from the hearing signers)
- keypointsD2.csv (data from the deaf signers)

## Importing the data set

We import two tables with the complete datasets for hearing signers and for deaf signers. The tables were previously created from OpenPose export files. In addition, the following cleaning up was done in advance:

- removing all keypoint data for keypoints with confidence level below 0.7. Note that after doing this, and also as we describe below, removing the first and last 20% of each video, 5 sentences ended up completely removed from the dataset.
- creating columns for signer ID and sentence ID

```
keypoints2 <- read.csv("keypoints2.csv", sep="\t") #the dataset for the hearing signers
keypointsD2 <- read.csv("keypointsD2.csv", sep="\t") #the dataset for the deaf signers
```

Columns in the dataset: file ID, file name, frame number in a file, signer ID, sentence ID, class (grammar and emotion), keypoint data from OpenPose in the format x, y, and c (confidence) for all keypoints on the face, for one keypoint on each of the hands (to determine utterance boundaries, see below). Note that the naming conventions are slightly different in the two datasets due to different settings when exporting output of OpenPose. These inconsistencies are corrected for in the code below.

## Measuring distance

The absolute y coordinates of eyebrow keypoints cannot be analysed directly because they simply show the position of the eyebrow relative to frame boundaries, which is primarily affected by the position of the body and the head of the signer in the frame. Instead, we will analyze distances between the eyebrow keypoints (keypoints 17 - 26) and the keypoint for the top of the nose (keypoint 27 in OpenPose). The distance is calculated using the usual formula from the x and y coordinates of two keypoints (d = sqrt((x1-x2)2+(y1-y2)2)), and is thus not affected by potential head and body movements.

```
keypoints2$rd17<-sqrt((keypoints2$y27-keypoints2$y17)^2 + (keypoints2$x27-keypoints2$x17)^2)
keypoints2$rd18<-sqrt((keypoints2$y27-keypoints2$y18)^2 + (keypoints2$x27-keypoints2$x18)^2)
keypoints2$rd19<-sqrt((keypoints2$y27-keypoints2$y19)^2 + (keypoints2$x27-keypoints2$x19)^2)
keypoints2$rd20<-sqrt((keypoints2$y27-keypoints2$y20)^2 + (keypoints2$x27-keypoints2$x20)^2)
keypoints2$rd21<-sqrt((keypoints2$y27-keypoints2$y21)^2 + (keypoints2$x27-keypoints2$x21)^2)
keypoints2$rd22<-sqrt((keypoints2$y27-keypoints2$y22)^2 + (keypoints2$x27-keypoints2$x22)^2)
keypoints2$rd23<-sqrt((keypoints2$y27-keypoints2$y23)^2 + (keypoints2$x27-keypoints2$x23)^2)
keypoints2$rd24<-sqrt((keypoints2$y27-keypoints2$y24)^2 + (keypoints2$x27-keypoints2$x24)^2)
keypoints2$rd25<-sqrt((keypoints2$y27-keypoints2$y25)^2 + (keypoints2$x27-keypoints2$x25)^2)
keypoints2$rd26<-sqrt((keypoints2$y27-keypoints2$y26)^2 + (keypoints2$x27-keypoints2$x26)^2)

keypointsD2$rd17<-sqrt((keypointsD2$face_y27-keypointsD2$face_y17)^2 + (keypointsD2$face_x27-keypointsD2$face_x17)^2)
keypointsD2$rd18<-sqrt((keypointsD2$face_y27-keypointsD2$face_y18)^2 + (keypointsD2$face_x27-keypointsD2$face_x18)^2)
keypointsD2$rd19<-sqrt((keypointsD2$face_y27-keypointsD2$face_y19)^2 + (keypointsD2$face_x27-keypointsD2$face_x19)^2)
keypointsD2$rd20<-sqrt((keypointsD2$face_y27-keypointsD2$face_y20)^2 + (keypointsD2$face_x27-keypointsD2$face_x20)^2)
keypointsD2$rd21<-sqrt((keypointsD2$face_y27-keypointsD2$face_y21)^2 + (keypointsD2$face_x27-keypointsD2$face_x21)^2)
keypointsD2$rd22<-sqrt((keypointsD2$face_y27-keypointsD2$face_y22)^2 + (keypointsD2$face_x27-keypointsD2$face_x22)^2)
keypointsD2$rd23<-sqrt((keypointsD2$face_y27-keypointsD2$face_y23)^2 + (keypointsD2$face_x27-keypointsD2$face_x23)^2)
keypointsD2$rd24<-sqrt((keypointsD2$face_y27-keypointsD2$face_y24)^2 + (keypointsD2$face_x27-keypointsD2$face_x24)^2)
keypointsD2$rd25<-sqrt((keypointsD2$face_y27-keypointsD2$face_y25)^2 + (keypointsD2$face_x27-keypointsD2$face_x25)^2)
keypointsD2$rd26<-sqrt((keypointsD2$face_y27-keypointsD2$face_y26)^2 + (keypointsD2$face_x27-keypointsD2$face_x26)^2)
```

## Trimming the videos

The videos were recorded in such a way that the signer starts with the hands lowered, and ends with the hands lowered. Because we consider the utterance to only start once signing has started, we wanted to remove the irrelevant preparatory and retraction parts of the recordings from the analysis.

First, we created a column with normalized frameID per video file (from 0 to 100 reflecting the percentage of file length in frames).

```
for (i in 1:359) {
keypoints2$fidnorm[keypoints2$video_id==i]<-keypoints2$frame_id[keypoints2$video_id==i]/max(keypoints2$frame_id[keypoints2$video_id==i])*100 }

for (i in 1:90) {
keypointsD2$fidnorm[keypointsD2$video_id==i]<-keypointsD2$frame_id[keypointsD2$video_id==i]/max(keypointsD2$frame_id[keypointsD2$video_id==i])*100 }
```

We then plotted the positions of the hands’ y coordinates for the normalized frame ID to determine the points at which hands would be raised and lowered on average (the code below is mostly commented out to avoid visual clutter).

```
plot(keypoints2$fidnorm[keypoints2$class=="statement-neutral"], keypoints2$hand_right_y0[keypoints2$class=="statement-neutral"])
lines(lowess(keypoints2$fidnorm[keypoints2$class=="statement-neutral"], keypoints2$hand_right_y0[keypoints2$class=="statement-neutral"]), col="red")
```

```
# 20 to 80 is reasonable

plot(keypoints2$fidnorm[keypoints2$class=="statement-anger"], keypoints2$hand_right_y0[keypoints2$class=="statement-anger"])
lines(lowess(keypoints2$fidnorm[keypoints2$class=="statement-anger"], keypoints2$hand_right_y0[keypoints2$class=="statement-anger"]), col="red")
```

```
# #clearly 20 to 80
# 
# plot(keypoints2$fidnorm[keypoints2$class=="statement-sur"], keypoints2$hand_right_y0[keypoints2$class=="statement-sur"])
# lines(lowess(keypoints2$fidnorm[keypoints2$class=="statement-sur"], keypoints2$hand_right_y0[keypoints2$class=="statement-sur"]), col="red")
# #clearly 20 to 80
# 
# plot(keypoints2$fidnorm[keypoints2$class=="general_question-neutral"], keypoints2$hand_right_y0[keypoints2$class=="general_question-neutral"])
# lines(lowess(keypoints2$fidnorm[keypoints2$class=="general_question-neutral"], keypoints2$hand_right_y0[keypoints2$class=="general_question-neutral"]), col="red") 
# # 20 to 80
# 
# plot(keypoints2$fidnorm[keypoints2$class=="general_question-anger"], keypoints2$hand_right_y0[keypoints2$class=="general_question-anger"])
# lines(lowess(keypoints2$fidnorm[keypoints2$class=="general_question-anger"], keypoints2$hand_right_y0[keypoints2$class=="general_question-anger"]), col="red")
# #clearly 20 to 80
# 
# plot(keypoints2$fidnorm[keypoints2$class=="general_question-sur"], keypoints2$hand_right_y0[keypoints2$class=="general_question-sur"])
# lines(lowess(keypoints2$fidnorm[keypoints2$class=="general_question-sur"], keypoints2$hand_right_y0[keypoints2$class=="general_question-sur"]), col="red")
# #clearly 20 to 80
# 
# plot(keypoints2$fidnorm[keypoints2$class=="partial_question-neutral"], keypoints2$hand_right_y0[keypoints2$class=="partial_question-neutral"])
# lines(lowess(keypoints2$fidnorm[keypoints2$class=="partial_question-neutral"], keypoints2$hand_right_y0[keypoints2$class=="partial_question-neutral"]), col="red") 
# # 15 to 85
# 
# plot(keypoints2$fidnorm[keypoints2$class=="partial_question-anger"], keypoints2$hand_right_y0[keypoints2$class=="partial_question-anger"])
# lines(lowess(keypoints2$fidnorm[keypoints2$class=="partial_question-anger"], keypoints2$hand_right_y0[keypoints2$class=="partial_question-anger"]), col="red")
# #clearly 20 to 80
# 
# plot(keypoints2$fidnorm[keypoints2$class=="partial_question-sur"], keypoints2$hand_right_y0[keypoints2$class=="partial_question-sur"])
# lines(lowess(keypoints2$fidnorm[keypoints2$class=="partial_question-sur"], keypoints2$hand_right_y0[keypoints2$class=="partial_question-sur"]), col="red")
# #clearly 20 to 80
# 
# plot(keypointsD2$fidnorm[keypointsD2$type=="st-neut"], keypointsD2$hand_right_y0[keypointsD2$type=="st-neut"])
# lines(lowess(keypointsD2$fidnorm[keypointsD2$type=="st-neut"], keypointsD2$hand_right_y0[keypointsD2$type=="st-neut"]), col="red") 
# # 20 to 80 is reasonable
# 
# plot(keypointsD2$fidnorm[keypointsD2$type=="st-anger"], keypointsD2$hand_right_y0[keypointsD2$type=="st-anger"])
# lines(lowess(keypointsD2$fidnorm[keypointsD2$type=="st-anger"], keypointsD2$hand_right_y0[keypointsD2$type=="st-anger"]), col="red")
# #clearly 20 to 80
# 
# plot(keypointsD2$fidnorm[keypointsD2$type=="st-sur"], keypointsD2$hand_right_y0[keypointsD2$type=="st-sur"])
# lines(lowess(keypointsD2$fidnorm[keypointsD2$type=="st-sur"], keypointsD2$hand_right_y0[keypointsD2$type=="st-sur"]), col="red")
# #clearly 20 to 80
# 
# plot(keypointsD2$fidnorm[keypointsD2$type=="gen-q-neut"], keypointsD2$hand_right_y0[keypointsD2$type=="gen-q-neut"])
# lines(lowess(keypointsD2$fidnorm[keypointsD2$type=="gen-q-neut"], keypointsD2$hand_right_y0[keypointsD2$type=="gen-q-neut"]), col="red") 
# # 20 to 80
# 
# plot(keypointsD2$fidnorm[keypointsD2$type=="gen-q-anger"], keypointsD2$hand_right_y0[keypointsD2$type=="gen-q-anger"])
# lines(lowess(keypointsD2$fidnorm[keypointsD2$type=="gen-q-anger"], keypointsD2$hand_right_y0[keypointsD2$type=="gen-q-anger"]), col="red")
# #clearly 20 to 80
# 
# plot(keypointsD2$fidnorm[keypointsD2$type=="gen-q-sur"], keypointsD2$hand_right_y0[keypointsD2$type=="gen-q-sur"])
# lines(lowess(keypointsD2$fidnorm[keypointsD2$type=="gen-q-sur"], keypointsD2$hand_right_y0[keypointsD2$type=="gen-q-sur"]), col="red")
# #more 10 to 75
# 
# plot(keypointsD2$fidnorm[keypointsD2$type=="part-q-neut"], keypointsD2$hand_right_y0[keypointsD2$type=="part-q-neut"])
# lines(lowess(keypointsD2$fidnorm[keypointsD2$type=="part-q-neut"], keypointsD2$hand_right_y0[keypointsD2$type=="part-q-neut"]), col="red") 
# # 20 to 80
# 
# plot(keypointsD2$fidnorm[keypointsD2$type=="part-q-anger"], keypointsD2$hand_right_y0[keypointsD2$type=="part-q-anger"])
# lines(lowess(keypointsD2$fidnorm[keypointsD2$type=="part-q-anger"], keypointsD2$hand_right_y0[keypointsD2$type=="part-q-anger"]), col="red")
# # 15 to 80
# 
# plot(keypointsD2$fidnorm[keypointsD2$type=="part-q-sur"], keypointsD2$hand_right_y0[keypointsD2$type=="part-q-sur"])
# lines(lowess(keypointsD2$fidnorm[keypointsD2$type=="part-q-sur"], keypointsD2$hand_right_y0[keypointsD2$type=="part-q-sur"]), col="red")
# #15 to 90
```

Based on the visual inspection, we decided to use 20% on both sides as cutoff points, and only include the data from the middle part for further analysis for all files.

```
keypoints3<-subset(keypoints2, fidnorm>=20&fidnorm<=80)
keypointsD3<-subset(keypointsD2, fidnorm>=20&fidnorm<=80)
```

We also create columns for average internal and external eyebrow positions by averaging between the two eyebrows in two points, one to represent the internal part, and one to represent the external part, because the two parts move partially independently. Note that the two eyebrows do not always move symmetrically, so we will separately analyze this asymmetry below, but for the main analysis we focus on the average between the two eyebrows.

```
keypoints3$yInternal<-(keypoints3$rd21+keypoints3$rd22)/2
keypoints3$yExternal<-(keypoints3$rd18+keypoints3$rd25)/2

keypointsD3$yInternal<-(keypointsD3$rd21+keypointsD3$rd22)/2
keypointsD3$yExternal<-(keypointsD3$rd18+keypointsD3$rd25)/2
```

Let’s also visually inspect the yInternal and yExternal in both sets for outliers:

```
hist(keypoints3$yInternal)
```

```
hist(keypoints3$yExternal)
```

```
hist(keypointsD3$yInternal)
```

```
hist(keypointsD3$yExternal)
```

We can see that there are clear outliers in the internal keypoints for both groups, and for external in the hearing group. Since those are clearly measurement errors (there could not be distances between eyebrow and nose ridge that large), we remove all the measurements above 50 for internal and 90 for external

```
keypoints3$yInternal[keypoints3$yInternal>50]<-NA
hist(keypoints3$yInternal)
```

```
keypoints3$yExternal[keypoints3$yExternal>90]<-NA
hist(keypoints3$yExternal)
```

```
keypointsD3$yInternal[keypointsD3$yInternal>50]<-NA
hist(keypointsD3$yInternal)
```

And we create columns for the two factors (sentence type and emotion) based on the signle column representing both of them now.

```
keypoints3$sType<-NA
keypoints3$sType[keypoints3$class=="general_question-anger"|keypoints3$class=="general_question-sur"|keypoints3$class=="general_question-neutral"]<-"GQ"
keypoints3$sType[keypoints3$class=="partial_question-anger"|keypoints3$class=="partial_question-sur"|keypoints3$class=="partial_question-neutral"]<-"PartQ"
keypoints3$sType[keypoints3$class=="statement-anger"|keypoints3$class=="statement-sur"|keypoints3$class=="statement-neutral"]<-"statement"

keypoints3$sType<-as.factor(keypoints3$sType)

keypoints3$emotion<-NA
keypoints3$emotion[keypoints3$class=="general_question-anger"|keypoints3$class=="partial_question-anger"|keypoints3$class=="statement-anger"]<-"anger"
keypoints3$emotion[keypoints3$class=="general_question-sur"|keypoints3$class=="partial_question-sur"|keypoints3$class=="statement-sur"]<-"sur"
 keypoints3$emotion[keypoints3$class=="general_question-neutral"|keypoints3$class=="partial_question-neutral"|keypoints3$class=="statement-neutral"]<-"neutral"
keypoints3$emotion<-as.factor(keypoints3$emotion)

keypointsD3$sType<-NA
keypointsD3$sType[keypointsD3$type=="gen-q-anger"|keypointsD3$type=="gen-q-sur"|keypointsD3$type=="gen-q-neut"]<-"GQ"
keypointsD3$sType[keypointsD3$type=="part-q-anger"|keypointsD3$type=="part-q-sur"|keypointsD3$type=="part-q-neut"]<-"PartQ"
keypointsD3$sType[keypointsD3$type=="st-anger"|keypointsD3$type=="st-sur"|keypointsD3$type=="st-neut"]<-"statement"

keypointsD3$sType<-as.factor(keypointsD3$sType)

keypointsD3$emotion<-NA
keypointsD3$emotion[keypointsD3$type=="gen-q-anger"|keypointsD3$type=="part-q-anger"|keypointsD3$type=="st-anger"]<-"anger"
keypointsD3$emotion[keypointsD3$type=="gen-q-sur"|keypointsD3$type=="part-q-sur"|keypointsD3$type=="st-sur"]<-"sur"
 keypointsD3$emotion[keypointsD3$type=="gen-q-neut"|keypointsD3$type=="part-q-neut"|keypointsD3$type=="st-neut"]<-"neutral"
keypointsD3$emotion<-as.factor(keypointsD3$emotion)
```

## Graphic inspection of the data

We explore the data by looking at boxplots showing interactions between the two factros under investigation (sentence type and emotion) for both groups of signers.

```
par(mfcol=c(1,2))

boxplot(yInternal ~ sType * emotion, data=keypoints3, ylab="Distance", xlab="Emotions", col=c("blue", "grey", "yellow"), xaxt="n", main = "Distance ~ sentence type * emotion  (hearing signers) \n Internal eyebrows") 
axis(1, 
     at = c(2,5,8), 
     labels = c("Anger", "Neutral", "Surprise"), 
     tick=FALSE)
legend("bottomleft", legend = c("General Question", "Wh-question", "Statement"), 
       fill=c("blue" , "grey", "yellow"), cex=0.5)

#boxplot(yInternal ~ emotion * sType, data=keypoints3) #another perspective


boxplot(yInternal ~ sType * emotion, data=keypointsD3, ylab="Distance", xlab="Emotions", col=c("blue", "grey", "yellow"), xaxt="n", main = "Distance ~ sentence type * emotion  (deaf signers) \n Internal eyebrows") 
axis(1, 
     at = c(2,5,8), 
     labels = c("Anger", "Neutral", "Surprise"), 
     tick=FALSE)
legend("bottomleft", legend = c("General Question", "Wh-question", "Statement"), 
       fill=c("blue" , "grey", "yellow"), cex=0.5)
```

```
#boxplot(yInternal ~ emotion * sType, data=keypointsD3) #another perspective

#Export at 1000px
 
boxplot(yExternal ~ sType * emotion, data=keypoints3, ylab="Distance", xlab="Emotions", col=c("blue", "grey", "yellow"), xaxt="n", main = "Distance ~ sentence type * emotion  (hearing signers) \n External eyebrows") 
axis(1, 
     at = c(2,5,8), 
     labels = c("Anger", "Neutral", "Surprise"), 
     tick=FALSE)
legend("bottomleft", legend = c("General Question", "Wh-question", "Statement"), 
       fill=c("blue" , "grey", "yellow"), cex=0.5)

#boxplot(yExternal ~ emotion * sType, data=keypoints3) #another perspective


boxplot(yExternal ~ sType * emotion, data=keypointsD3, ylab="Distance", xlab="Emotions", col=c("blue", "grey", "yellow"), xaxt="n", main = "Distance ~ sentence type * emotion  (deaf signers) \n External eyebrows") 
axis(1, 
     at = c(2,5,8), 
     labels = c("Anger", "Neutral", "Surprise"), 
     tick=FALSE)
legend("bottomleft", legend = c("General Question", "Wh-question", "Statement"), 
       fill=c("blue" , "grey", "yellow"), cex=0.5)
```

```
#boxplot(yExternal ~ emotion * sType, data=keypointsD3) #another perspective


par(mfcol=c(1,1))
```

For internal eyebrows, deaf signers show an expected neat pattern: general question > wh-questions > statements, and also with expected hierarchy of emotions (surprise > neutral > anger), with somewhat less differences between sentence types when emotions are present.

The hearing signers show a more complicated pattern: while general questions always have higher distance, wh-questions and statements are not consistent and depend on emotions. Also while anger lowers eyebrows consistently, surprise is less consistent.

External eyebrows in deaf signers follow the expected pattern but with small differences between conditions (both sentence types and emotions). For the hearing signers, a complex pattern is observed, with general question having a consistent effect, but wh-questions and statements behaving in interaction with emotions.

In general, it is clear that the two groups are not identical, and that there are complex interactions between emotions and sentence types, although the main effects are in the expected direction.

## Statistical analysis

We can now build a model to see which factors contribute to eyebrow position to what extent, also taking into account individual effects of signers and sentences.

We combine the relevant part of the deaf and hearing datasets in one table.

```
keypoints.all<-data.frame(keypoints3$yInternal, keypoints3$yExternal, keypoints3$sentenceID, keypoints3$signerID, keypoints3$sType, keypoints3$emotion)

keypoints.all$group<-"hearing"
colnames(keypoints.all)<-c("yInternal", "yExternal", "sentence", "signer", "sType", "emotion", "group")

keypoints.2<-data.frame(keypointsD3$yInternal, keypointsD3$yExternal, keypointsD3$sentence, keypointsD3$speaker_id, keypointsD3$sType, keypointsD3$emotion)

keypoints.2$group<-"deaf"

colnames(keypoints.2)<-c("yInternal", "yExternal", "sentence", "signer", "sType", "emotion", "group")


keypoints.all<-rbind(keypoints.all, keypoints.2)

#the levels for sentences are not the same in the two datasets, so we change this
keypoints.all$sentence[keypoints.all$sentence=="dev_upala"]<-"devupala"
keypoints.all$sentence[keypoints.all$sentence=="dom_post"]<-"dom.postroili"
keypoints.all$sentence[keypoints.all$sentence=="mal_chit"]<-"mal.chit"
keypoints.all$sentence[keypoints.all$sentence=="mama_ust"]<-"mama.ustala"
keypoints.all$sentence[keypoints.all$sentence=="okno_razb"]<-"okno.razb"
keypoints.all$sentence[keypoints.all$sentence=="papa_beg"]<-"papa.bezhit"
keypoints.all$sentence[keypoints.all$sentence=="reb_tanc"]<-"reb.tanc"
keypoints.all$sentence[keypoints.all$sentence=="sob_est"]<-"sobaka.est"
keypoints.all$sentence[keypoints.all$sentence=="tel_slom"]<-"tel.slom"
keypoints.all$sentence[keypoints.all$sentence=="uchit_smeh"]<-"uch.smeh"
keypoints.all$sentence<-droplevels(keypoints.all$sentence)
```

We have separate columns for the average relative internal eyebrow position (between the two brows), and a separate column for external. We will model them in two separate models.

### Internal eyebrow position

First we create a dataset for the internal keypoints. We select only the relevant columns, and then we aggregate the table so that there is now only one measurement per every file. We do this because we do not want to analyze the dynamics of eyebrow movement within each file, as this would require a much more sophisticated model.

```
internal<-aggregate(keypoints.all$yInternal, by=list(keypoints.all$signer, keypoints.all$sentence, keypoints.all$sType, keypoints.all$emotion, keypoints.all$group), mean)
colnames(internal)<-c('speaker_id', 'sentence', 'sType', 'emotion', 'group', 'x')
internal<-na.omit(internal)
```

We need to also need to inspect the values of yInternal to see if there are any unreasonable outliers. We do not see anything completely out of the ordinary here, so we can model it.

```
hist(internal$x)
```

We set up orthogonal contrasts for our factors: we compare neutral to anger, and surprise to the average of neutral and anger, and we compare wh-questions to statements, and their average to polar questions.

```
contrast <- cbind (c(-1/2, 1/2, 0), c(-1/3, -1/3, 2/3))   # ang neut surpr
colnames (contrast) <- c("+neutral-anger", "+surprise")
contrasts (internal$emotion) <- contrast
contrasts (internal$emotion)
```

```
##         +neutral-anger  +surprise
## anger             -0.5 -0.3333333
## neutral            0.5 -0.3333333
## sur                0.0  0.6666667
```

```
contrast <- cbind (c(0, 1/2, -1/2), c(2/3, -1/3, -1/3))   # gq partQ statement
colnames (contrast) <- c("+wh-st", "+polar")
contrasts (internal$sType) <- contrast
contrasts (internal$sType)
```

```
##           +wh-st     +polar
## GQ           0.0  0.6666667
## PartQ        0.5 -0.3333333
## statement   -0.5 -0.3333333
```

```
internal$group<-as.factor(internal$group)


contrast <- cbind (c(1/2, -1/2)) #deaf, hearing
colnames (contrast) <- c("+deaf")
contrasts (internal$group) <- contrast
contrasts(internal$group)
```

```
##         +deaf
## deaf      0.5
## hearing  -0.5
```

We build the model where yInternal is predicted by emotion, sentence type, group, and all possible interactions, as well as random intercepts and slopes for emotion and group by sentence, and for emotion and sentence type by speaker.

Because models with 5 or less levels of a random factor often result in singular fits, we use a prior over the covarience matrix of random effects using the *blme* package.

```
library(blme) #bayesian model with covert prior for covariance matrix
```

```
## Loading required package: lme4
```

```
## Loading required package: Matrix
```

```
library(optimx) #optimizer

modelIntb<-blmer(x ~ emotion * sType * group + (emotion*group|sentence) + (emotion*sType|speaker_id), data=internal, control = lmerControl(optimizer ='optimx', optCtrl=list(method='nlminb'))) #
summary(modelIntb)
```

```
## Cov prior  : sentence ~ wishart(df = 8.5, scale = Inf, posterior.scale = cov, common.scale = TRUE)
##            : speaker_id ~ wishart(df = 11.5, scale = Inf, posterior.scale = cov, common.scale = TRUE)
## Prior dev  : 39.1937
## 
## Linear mixed model fit by REML ['blmerMod']
## Formula: x ~ emotion * sType * group + (emotion * group | sentence) +  
##     (emotion * sType | speaker_id)
##    Data: internal
## Control: lmerControl(optimizer = "optimx", optCtrl = list(method = "nlminb"))
## 
## REML criterion at convergence: 3510.5
## 
## Scaled residuals: 
##     Min      1Q  Median      3Q     Max 
## -3.2289 -0.6237 -0.0516  0.5680  4.8770 
## 
## Random effects:
##  Groups     Name                              Variance Std.Dev. Corr       
##  sentence   (Intercept)                       0.4159   0.6449              
##             emotion+neutral-anger             0.4729   0.6877    0.51      
##             emotion+surprise                  0.3913   0.6256   -0.07  0.42
##             group+deaf                        0.3494   0.5911    0.14 -0.16
##             emotion+neutral-anger:group+deaf  0.8009   0.8949    0.25 -0.40
##             emotion+surprise:group+deaf       0.5544   0.7446   -0.33  0.06
##  speaker_id (Intercept)                       4.6280   2.1513              
##             emotion+neutral-anger             5.5603   2.3580    0.38      
##             emotion+surprise                  3.0022   1.7327    0.68  0.76
##             sType+wh-st                       0.9797   0.9898   -0.38 -0.44
##             sType+polar                       0.5054   0.7109    0.30 -0.03
##             emotion+neutral-anger:sType+wh-st 1.7495   1.3227   -0.56 -0.24
##             emotion+surprise:sType+wh-st      2.6741   1.6353   -0.20 -0.37
##             emotion+neutral-anger:sType+polar 7.9959   2.8277    0.28  0.41
##             emotion+surprise:sType+polar      2.4972   1.5803   -0.82 -0.45
##  Residual                                     3.8860   1.9713              
##                                     
##                                     
##                                     
##                                     
##  -0.52                              
##  -0.61  0.44                        
##  -0.12  0.33 -0.09                  
##                                     
##                                     
##                                     
##  -0.41                              
##   0.21 -0.19                        
##  -0.33  0.44 -0.08                  
##  -0.46  0.29  0.03  0.05            
##   0.62 -0.59  0.40 -0.15 -0.73      
##  -0.60  0.10  0.00  0.32  0.06  0.03
##                                     
## Number of obs: 802, groups:  sentence, 10; speaker_id, 9
## 
## Fixed effects:
##                                              Estimate Std. Error t value
## (Intercept)                                   27.0433     0.7531  35.910
## emotion+neutral-anger                          3.7403     0.8380   4.463
## emotion+surprise                               4.1729     0.6317   6.606
## sType+wh-st                                    0.3004     0.3736   0.804
## sType+polar                                    2.3132     0.2812   8.227
## group+deaf                                     0.7186     1.4619   0.492
## emotion+neutral-anger:sType+wh-st              0.1893     0.6107   0.310
## emotion+surprise:sType+wh-st                  -1.5176     0.6580  -2.306
## emotion+neutral-anger:sType+polar              1.0998     1.0159   1.083
## emotion+surprise:sType+polar                  -0.3879     0.6174  -0.628
## emotion+neutral-anger:group+deaf              -1.9871     1.6431  -1.209
## emotion+surprise:group+deaf                    0.6453     1.2227   0.528
## sType+wh-st:group+deaf                         1.2128     0.7472   1.623
## sType+polar:group+deaf                        -1.7582     0.5623  -3.127
## emotion+neutral-anger:sType+wh-st:group+deaf   1.7112     1.2215   1.401
## emotion+surprise:sType+wh-st:group+deaf        3.0188     1.3161   2.294
## emotion+neutral-anger:sType+polar:group+deaf  -1.8485     2.0319  -0.910
## emotion+surprise:sType+polar:group+deaf       -1.1640     1.2349  -0.943
```

```
## 
## Correlation matrix not shown by default, as p = 18 > 12.
## Use print(x, correlation=TRUE)  or
##     vcov(x)        if you need it
```

```
library("car")
```

```
## Loading required package: carData
```

```
## Registered S3 methods overwritten by 'car':
##   method                          from
##   influence.merMod                lme4
##   cooks.distance.influence.merMod lme4
##   dfbeta.influence.merMod         lme4
##   dfbetas.influence.merMod        lme4
```

```
Anova(modelIntb)
```

```
## Analysis of Deviance Table (Type II Wald chisquare tests)
## 
## Response: x
##                       Chisq Df Pr(>Chisq)    
## emotion             68.9030  2  1.091e-15 ***
## sType               78.3283  2  < 2.2e-16 ***
## group                1.6090  1  0.2046389    
## emotion:sType        5.1383  4  0.2733975    
## emotion:group        5.3615  2  0.0685106 .  
## sType:group         15.5812  2  0.0004136 ***
## emotion:sType:group  9.3184  4  0.0536153 .  
## ---
## Signif. codes:  0 '***' 0.001 '**' 0.01 '*' 0.05 '.' 0.1 ' ' 1
```

```
#coefficients(summary(modelIntb))[4,1]
```

Anova shows that there are significant effects of emotion, sentence type, and a significant interaction of sentence type and group. Looking more specifically at the individual effects from the model and using the t-values as rough estimation of significance, we can see the following:

- Anger lowers eyebrows in comparison to neutral (3.7402722 px, t=4.4633749)
- Surprise raises eyebrows in comparison to the average between anger and neutral (4.1728803 px, , t=6.6058745)
- Polar question raises eyebrows in comparison to the other two types (2.3131597 px, t=8.2274386)
- The effect of polar questions is lower in deaf signers than in hearing signers (-1.758173 px, , t=-3.1267246)

Let’s also graphically represent the predicted values for the internal eyebrow positions:

```
internal$predicted<-predict(modelIntb)
boxplot(predicted~ sType * emotion  * group, data=internal, las=2, xlab=" ")
```

### External eyebrow position

First we create a dataset for the external keypoints. We select only the relevant columns, and then we aggregate the table so that there is now only one measurement per every file. We do this because we do not want to analyze the dynamics of eyebrow movement within each file, as this would require a much more sophisticated model.

```
external<-aggregate(keypoints.all$yExternal, by=list(keypoints.all$signer, keypoints.all$sentence, keypoints.all$sType, keypoints.all$emotion, keypoints.all$group), mean)
colnames(external)<-c('speaker_id', 'sentence', 'sType', 'emotion', 'group', 'x')
external<-na.omit(external)
```

We need to also need to inspect the values of yExternal to see if there are any unreasonable outliers. We do not see anything completely out of the ordinary here, so we can model it.

```
hist(external$x)
```

We set up orthogonal contrasts for our factors: we compare neutral to anger, and surprise to the average of neutral and anger, and we compare wh-questions to statements, and their average to polar questions.

```
contrast <- cbind (c(-1/2, 1/2, 0), c(-1/3, -1/3, 2/3))   # ang neut surpr
colnames (contrast) <- c("+neutral-anger", "+surprise")
contrasts (external$emotion) <- contrast
contrasts (external$emotion)
```

```
##         +neutral-anger  +surprise
## anger             -0.5 -0.3333333
## neutral            0.5 -0.3333333
## sur                0.0  0.6666667
```

```
contrast <- cbind (c(0, 1/2, -1/2), c(2/3, -1/3, -1/3))   # gq partQ statement
colnames (contrast) <- c("+wh-st", "+polar")
contrasts (external$sType) <- contrast
contrasts (external$sType)
```

```
##           +wh-st     +polar
## GQ           0.0  0.6666667
## PartQ        0.5 -0.3333333
## statement   -0.5 -0.3333333
```

```
external$group<-as.factor(external$group)


contrast <- cbind (c(1/2, -1/2)) #deaf, hearing
colnames (contrast) <- c("+deaf")
contrasts (external$group) <- contrast
contrasts(external$group)
```

```
##         +deaf
## deaf      0.5
## hearing  -0.5
```

We build the model where yExternal is predicted by emotion, sentence type, group, and all possible interactions, as well as random intercepts and slopes for emotion and group by sentence, and for emotion and sentence type by speaker.

Because models with 5 or less levels of a random factor often result in singular fits, we use a prior over the covarience matrix of random effects using the *blme* package.

```
library(blme) #bayesian model with covert prior for covariance matrix
library(optimx) #optimizer

modelExtb<-blmer(x ~ emotion * sType * group + (emotion*group|sentence) + (emotion*sType|speaker_id), data=external, control = lmerControl(optimizer ='optimx', optCtrl=list(method='nlminb'))) #
summary(modelExtb)
```

```
## Cov prior  : sentence ~ wishart(df = 8.5, scale = Inf, posterior.scale = cov, common.scale = TRUE)
##            : speaker_id ~ wishart(df = 11.5, scale = Inf, posterior.scale = cov, common.scale = TRUE)
## Prior dev  : 45.1841
## 
## Linear mixed model fit by REML ['blmerMod']
## Formula: x ~ emotion * sType * group + (emotion * group | sentence) +  
##     (emotion * sType | speaker_id)
##    Data: external
## Control: lmerControl(optimizer = "optimx", optCtrl = list(method = "nlminb"))
## 
## REML criterion at convergence: 3827
## 
## Scaled residuals: 
##     Min      1Q  Median      3Q     Max 
## -4.6714 -0.5929 -0.0180  0.5316  3.8823 
## 
## Random effects:
##  Groups     Name                              Variance Std.Dev. Corr       
##  sentence   (Intercept)                       1.3486   1.1613              
##             emotion+neutral-anger             0.2342   0.4840    0.59      
##             emotion+surprise                  0.5415   0.7359   -0.26 -0.32
##             group+deaf                        2.2964   1.5154    0.68  0.67
##             emotion+neutral-anger:group+deaf  0.4726   0.6875   -0.15  0.00
##             emotion+surprise:group+deaf       0.3668   0.6056   -0.17 -0.02
##  speaker_id (Intercept)                       9.6830   3.1118              
##             emotion+neutral-anger             5.2311   2.2872    0.05      
##             emotion+surprise                  1.2472   1.1168   -0.33  0.64
##             sType+wh-st                       0.9665   0.9831    0.20 -0.56
##             sType+polar                       1.2106   1.1003   -0.26 -0.12
##             emotion+neutral-anger:sType+wh-st 4.4411   2.1074   -0.78  0.36
##             emotion+surprise:sType+wh-st      1.0310   1.0154   -0.11 -0.33
##             emotion+neutral-anger:sType+polar 4.6957   2.1669   -0.08 -0.09
##             emotion+surprise:sType+polar      1.9507   1.3967   -0.01 -0.08
##  Residual                                     5.8040   2.4091              
##                                     
##                                     
##                                     
##                                     
##  -0.74                              
##  -0.27  0.17                        
##  -0.18  0.02  0.18                  
##                                     
##                                     
##                                     
##  -0.42                              
##   0.37 -0.30                        
##   0.55 -0.18  0.02                  
##  -0.37  0.34 -0.31  0.00            
##   0.12 -0.59  0.57 -0.19 -0.37      
##  -0.10 -0.43  0.41 -0.20 -0.42  0.68
##                                     
## Number of obs: 804, groups:  sentence, 10; speaker_id, 9
## 
## Fixed effects:
##                                              Estimate Std. Error t value
## (Intercept)                                   63.2685     1.1097  57.012
## emotion+neutral-anger                          0.4497     0.8098   0.555
## emotion+surprise                               2.4578     0.4769   5.154
## sType+wh-st                                    0.0860     0.3905   0.220
## sType+polar                                    2.4809     0.4113   6.031
## group+deaf                                    -1.8585     2.1486  -0.865
## emotion+neutral-anger:sType+wh-st             -0.7024     0.8730  -0.805
## emotion+surprise:sType+wh-st                  -1.9583     0.5596  -3.499
## emotion+neutral-anger:sType+polar              1.1417     0.8521   1.340
## emotion+surprise:sType+polar                   0.4769     0.6068   0.786
## emotion+neutral-anger:group+deaf              -1.7525     1.6052  -1.092
## emotion+surprise:group+deaf                    0.1255     0.8542   0.147
## sType+wh-st:group+deaf                         1.8247     0.7811   2.336
## sType+polar:group+deaf                        -2.3010     0.8227  -2.797
## emotion+neutral-anger:sType+wh-st:group+deaf   0.2305     1.7460   0.132
## emotion+surprise:sType+wh-st:group+deaf        3.3201     1.1193   2.966
## emotion+neutral-anger:sType+polar:group+deaf  -1.2815     1.7043  -0.752
## emotion+surprise:sType+polar:group+deaf       -2.1668     1.2135  -1.786
```

```
## 
## Correlation matrix not shown by default, as p = 18 > 12.
## Use print(x, correlation=TRUE)  or
##     vcov(x)        if you need it
```

```
library("car")
Anova(modelExtb)
```

```
## Analysis of Deviance Table (Type II Wald chisquare tests)
## 
## Response: x
##                       Chisq Df Pr(>Chisq)    
## emotion             32.3529  2  9.433e-08 ***
## sType               33.9414  2  4.263e-08 ***
## group                1.4486  1    0.22875    
## emotion:sType       11.2735  4    0.02366 *  
## emotion:group        1.5849  2    0.45274    
## sType:group          8.9346  2    0.01148 *  
## emotion:sType:group 10.4573  4    0.03339 *  
## ---
## Signif. codes:  0 '***' 0.001 '**' 0.01 '*' 0.05 '.' 0.1 ' ' 1
```

The results are different here. Anova shows significant effects of emotion, sentence type, a significant interaction of emotion and sentence type, and a significant interaction of sentence type and group, and a significant three-way interaction. Looking at specific effects in the regression model, we can make the following observations:

- Anger does not significantly affect the external eyebrow height
- Surprise raises eyebrows in comparison to the average of neutral and anger (2.4578352 px, t=5.1540089)
- Polar questions raise eyebrows in comparison to the other two types (2.4808955 px, t=6.0312189)
- The effect of surprise is less for wh-questions (-1.958327 px, t=-3.4992257). Because of this, the difference between wh-questions and statements is more visible with surprise
- The effect of polar questions is less for the deaf group (-2.3009732 px, t=-2.7968974), but the effect of wh-questions in higher (1.8246586 px, t=2.3360712).
- The effect of surprise on wh-questions is higher for the deaf group (3.3201255 px, t=2.9662739). What this basically means is that wh-questions are not different from statements with surprise in this group, while they are different in the hearing group.

Let’s also graphically represent the predicted values for the external eyebrow positions:

```
external$predicted<-predict(modelExtb)
boxplot(predicted~ sType * emotion  * group, data=external, las=2, xlab=" ")
```

From this it is clear that in both deaf and hearing groups emotions are different from each other, and which each emotion there are differences between sentence types (sometimes mediated by emotions). In the deaf group, the differences overall are smaller, but also the variation is smaller.

## Individual models for hearing and deaf

Given that there are differences between groups, it would be interesting to look at the two groups separately to see whether the other effects remain. We first do it for the deaf group.

### Deaf signers only

We select the deaf signers only, internal eyebrows.

```
internalD<-subset(internal, group=="deaf")
```

We need to also need to inspect the values of yInternal to see if there are any unreasonable outliers. We do not see anything completely out of the ordinary here, so we can model it.

```
hist(internalD$x)
```

We set up orthogonal contrasts for our factors: we compare neutral to anger, and surprise to the average of neutral and anger, and we compare wh-questions to statements, and their average to polar questions.

```
contrast <- cbind (c(-1/2, 1/2, 0), c(-1/3, -1/3, 2/3))   # ang neut surpr
colnames (contrast) <- c("+neutral-anger", "+surprise")
contrasts (internalD$emotion) <- contrast
contrasts (internalD$emotion)
```

```
##         +neutral-anger  +surprise
## anger             -0.5 -0.3333333
## neutral            0.5 -0.3333333
## sur                0.0  0.6666667
```

```
contrast <- cbind (c(0, 1/2, -1/2), c(2/3, -1/3, -1/3))   # gq partQ statement
colnames (contrast) <- c("+wh-st", "+polar")
contrasts (internalD$sType) <- contrast
contrasts (internalD$sType)
```

```
##           +wh-st     +polar
## GQ           0.0  0.6666667
## PartQ        0.5 -0.3333333
## statement   -0.5 -0.3333333
```

We build the model where yInternal is predicted by emotion, sentence type, group, and all possible interactions, as well as random intercepts and slopes for emotion and group by sentence, and for emotion and sentence type by speaker.

Because models with 5 or less levels of a random factor often result in singular fits, we use a prior over the covarience matrix of random effects using the *blme* package.

```
library(blme) #bayesian model with covert prior for covariance matrix
library(optimx) #optimizer

modelIntbD<-blmer(x ~ emotion * sType + (emotion|sentence) + (emotion*sType|speaker_id), data=internalD, control = lmerControl(optimizer ='optimx', optCtrl=list(method='nlminb'))) #
summary(modelIntbD)
```

```
## Cov prior  : sentence ~ wishart(df = 5.5, scale = Inf, posterior.scale = cov, common.scale = TRUE)
##            : speaker_id ~ wishart(df = 11.5, scale = Inf, posterior.scale = cov, common.scale = TRUE)
## Prior dev  : 29.9713
## 
## Linear mixed model fit by REML ['blmerMod']
## Formula: x ~ emotion * sType + (emotion | sentence) + (emotion * sType |  
##     speaker_id)
##    Data: internalD
## Control: lmerControl(optimizer = "optimx", optCtrl = list(method = "nlminb"))
## 
## REML criterion at convergence: 1957
## 
## Scaled residuals: 
##     Min      1Q  Median      3Q     Max 
## -2.4974 -0.6435 -0.0560  0.6089  4.7158 
## 
## Random effects:
##  Groups     Name                              Variance Std.Dev. Corr       
##  sentence   (Intercept)                       0.5547   0.7448              
##             emotion+neutral-anger             0.3822   0.6182    0.68      
##             emotion+surprise                  0.4159   0.6449   -0.34 -0.03
##  speaker_id (Intercept)                       3.0482   1.7459              
##             emotion+neutral-anger             2.2000   1.4832   -0.35      
##             emotion+surprise                  1.8781   1.3704    0.27  0.17
##             sType+wh-st                       1.0419   1.0208   -0.56 -0.16
##             sType+polar                       1.2162   1.1028    0.61 -0.26
##             emotion+neutral-anger:sType+wh-st 4.6200   2.1494   -0.45 -0.50
##             emotion+surprise:sType+wh-st      2.7876   1.6696    0.80 -0.43
##             emotion+neutral-anger:sType+polar 3.5039   1.8719   -0.07  0.38
##             emotion+surprise:sType+polar      1.3913   1.1796   -0.53  0.63
##  Residual                                     3.9599   1.9900              
##                                     
##                                     
##                                     
##                                     
##                                     
##                                     
##                                     
##   0.08                              
##   0.26  0.03                        
##  -0.34  0.64 -0.11                  
##   0.12 -0.53  0.31 -0.30            
##   0.57  0.35  0.42 -0.16 -0.30      
##   0.02  0.25 -0.14 -0.06 -0.59  0.42
##                                     
## Number of obs: 446, groups:  sentence, 10; speaker_id, 5
## 
## Fixed effects:
##                                    Estimate Std. Error t value
## (Intercept)                       27.401962   0.820965  33.378
## emotion+neutral-anger              2.742696   0.729242   3.761
## emotion+surprise                   4.496504   0.676017   6.651
## sType+wh-st                        0.906061   0.511644   1.771
## sType+polar                        1.435465   0.532150   2.697
## emotion+neutral-anger:sType+wh-st  1.047246   1.116294   0.938
## emotion+surprise:sType+wh-st      -0.007044   0.892436  -0.008
## emotion+neutral-anger:sType+polar  0.180241   0.970303   0.186
## emotion+surprise:sType+polar      -0.971934   0.676196  -1.437
## 
## Correlation of Fixed Effects:
##             (Intr) emtn+- emtn+s sTyp+- sTyp+p e+-:T+- e+:T+- em+-:T+
## emtn+ntrl-n -0.254                                                   
## emotn+srprs  0.201  0.136                                            
## sType+wh-st -0.477 -0.133  0.069                                     
## sType+polar  0.540 -0.217  0.216  0.028                              
## emtn+n-:T+- -0.368 -0.395 -0.270  0.494 -0.091                       
## emtn+sr:T+-  0.639 -0.326  0.092 -0.397  0.242 -0.215                
## emtn+nt-:T+ -0.059  0.301  0.447  0.270  0.334 -0.115  -0.216        
## emtn+srp:T+ -0.396  0.446  0.013  0.170 -0.100 -0.041  -0.382  0.284
```

```
library("car")
Anova(modelIntbD)
```

```
## Analysis of Deviance Table (Type II Wald chisquare tests)
## 
## Response: x
##                 Chisq Df Pr(>Chisq)    
## emotion       97.4404  2  < 2.2e-16 ***
## sType         10.1000  2   0.006409 ** 
## emotion:sType  3.4417  4   0.486806    
## ---
## Signif. codes:  0 '***' 0.001 '**' 0.01 '*' 0.05 '.' 0.1 ' ' 1
```

We still observe the significant effects of both emotions in expected direcitons and of polar questions, but not of wh-questions.

We select the deaf signers only, external eyebrows.

```
externalD<-subset(external, group=="deaf")
```

We need to also need to inspect the values of yInternal to see if there are any unreasonable outliers. We do not see anything completely out of the ordinary here, so we can model it.

```
hist(externalD$x)
```

We set up orthogonal contrasts for our factors: we compare neutral to anger, and surprise to the average of neutral and anger, and we compare wh-questions to statements, and their average to polar questions.

```
contrast <- cbind (c(-1/2, 1/2, 0), c(-1/3, -1/3, 2/3))   # ang neut surpr
colnames (contrast) <- c("+neutral-anger", "+surprise")
contrasts (externalD$emotion) <- contrast
contrasts (externalD$emotion)
```

```
##         +neutral-anger  +surprise
## anger             -0.5 -0.3333333
## neutral            0.5 -0.3333333
## sur                0.0  0.6666667
```

```
contrast <- cbind (c(0, 1/2, -1/2), c(2/3, -1/3, -1/3))   # gq partQ statement
colnames (contrast) <- c("+wh-st", "+polar")
contrasts (externalD$sType) <- contrast
contrasts (externalD$sType)
```

```
##           +wh-st     +polar
## GQ           0.0  0.6666667
## PartQ        0.5 -0.3333333
## statement   -0.5 -0.3333333
```

We build the model where yExternal is predicted by emotion, sentence type, group, and all possible interactions, as well as random intercepts and slopes for emotion and group by sentence, and for emotion and sentence type by speaker.

Because models with 5 or less levels of a random factor often result in singular fits, we use a prior over the covarience matrix of random effects using the *blme* package.

```
library(blme) #bayesian model with covert prior for covariance matrix
library(optimx) #optimizer

modelExtbD<-blmer(x ~ emotion * sType + (emotion|sentence) + (emotion*sType|speaker_id), data=externalD, control = lmerControl(optimizer ='optimx', optCtrl=list(method='nlminb'))) #
summary(modelExtbD)
```

```
## Cov prior  : sentence ~ wishart(df = 5.5, scale = Inf, posterior.scale = cov, common.scale = TRUE)
##            : speaker_id ~ wishart(df = 11.5, scale = Inf, posterior.scale = cov, common.scale = TRUE)
## Prior dev  : 31.1726
## 
## Linear mixed model fit by REML ['blmerMod']
## Formula: x ~ emotion * sType + (emotion | sentence) + (emotion * sType |  
##     speaker_id)
##    Data: externalD
## Control: lmerControl(optimizer = "optimx", optCtrl = list(method = "nlminb"))
## 
## REML criterion at convergence: 2226.9
## 
## Scaled residuals: 
##     Min      1Q  Median      3Q     Max 
## -4.1942 -0.6316 -0.0491  0.5525  3.4978 
## 
## Random effects:
##  Groups     Name                              Variance Std.Dev. Corr       
##  sentence   (Intercept)                        3.0883  1.7574              
##             emotion+neutral-anger              0.3453  0.5877    0.54      
##             emotion+surprise                   0.4536  0.6735   -0.59 -0.29
##  speaker_id (Intercept)                       20.8711  4.5685              
##             emotion+neutral-anger              5.0844  2.2549    0.20      
##             emotion+surprise                   1.2340  1.1109   -0.48  0.58
##             sType+wh-st                        0.3179  0.5639    0.13 -0.22
##             sType+polar                        1.7504  1.3230   -0.31 -0.14
##             emotion+neutral-anger:sType+wh-st  9.1879  3.0312   -0.80  0.21
##             emotion+surprise:sType+wh-st       2.5643  1.6013   -0.12 -0.57
##             emotion+neutral-anger:sType+polar  2.2466  1.4989   -0.02 -0.32
##             emotion+surprise:sType+polar       1.5968  1.2636    0.07  0.54
##  Residual                                      7.1926  2.6819              
##                                     
##                                     
##                                     
##                                     
##                                     
##                                     
##                                     
##  -0.25                              
##   0.25  0.01                        
##   0.69 -0.21  0.19                  
##  -0.46  0.14 -0.07 -0.16            
##  -0.11  0.07  0.01 -0.05  0.01      
##   0.44 -0.13  0.12  0.17 -0.42 -0.10
##                                     
## Number of obs: 447, groups:  sentence, 10; speaker_id, 5
## 
## Fixed effects:
##                                   Estimate Std. Error t value
## (Intercept)                        62.3387     2.1211  29.389
## emotion+neutral-anger              -0.4310     1.0716  -0.402
## emotion+surprise                    2.5214     0.6036   4.177
## sType+wh-st                         0.9987     0.4003   2.495
## sType+polar                         1.3333     0.6500   2.051
## emotion+neutral-anger:sType+wh-st  -0.5860     1.5555  -0.377
## emotion+surprise:sType+wh-st       -0.2989     0.9726  -0.307
## emotion+neutral-anger:sType+polar   0.5013     0.9411   0.533
## emotion+surprise:sType+polar       -0.6108     0.8026  -0.761
## 
## Correlation of Fixed Effects:
##             (Intr) emtn+- emtn+s sTyp+- sTyp+p e+-:T+- e+:T+- em+-:T+
## emtn+ntrl-n  0.203                                                   
## emotn+srprs -0.439  0.432                                            
## sType+wh-st  0.079 -0.133 -0.130                                     
## sType+polar -0.272 -0.121  0.184  0.008                              
## emtn+n-:T+- -0.675  0.171  0.492 -0.110  0.152                       
## emtn+sr:T+- -0.083 -0.396 -0.280  0.060 -0.049 -0.107                
## emtn+nt-:T+ -0.016 -0.218 -0.060  0.031  0.007 -0.030   0.008        
## emtn+srp:T+  0.046  0.362  0.255 -0.060  0.078  0.106  -0.220 -0.051
```

```
library("car")
Anova(modelExtbD)
```

```
## Analysis of Deviance Table (Type II Wald chisquare tests)
## 
## Response: x
##                 Chisq Df Pr(>Chisq)    
## emotion       32.5104  2  8.719e-08 ***
## sType         10.3882  2   0.005549 ** 
## emotion:sType  1.1667  4   0.883548    
## ---
## Signif. codes:  0 '***' 0.001 '**' 0.01 '*' 0.05 '.' 0.1 ' ' 1
```

Here we still see the expected effects of surprise, and the effect of polar questions, and even of wh-questions, which is significant but low.

### Hearing signers only

We select the hearing signers only, internal eyebrows.

```
internalH<-subset(internal, group=="hearing")
```

We need to also need to inspect the values of yInternal to see if there are any unreasonable outliers. We do not see anything completely out of the ordinary here, so we can model it.

```
hist(internalH$x)
```

We set up orthogonal contrasts for our factors: we compare neutral to anger, and surprise to the average of neutral and anger, and we compare wh-questions to statements, and their average to polar questions.

```
contrast <- cbind (c(-1/2, 1/2, 0), c(-1/3, -1/3, 2/3))   # ang neut surpr
colnames (contrast) <- c("+neutral-anger", "+surprise")
contrasts (internalH$emotion) <- contrast
contrasts (internalH$emotion)
```

```
##         +neutral-anger  +surprise
## anger             -0.5 -0.3333333
## neutral            0.5 -0.3333333
## sur                0.0  0.6666667
```

```
contrast <- cbind (c(0, 1/2, -1/2), c(2/3, -1/3, -1/3))   # gq partQ statement
colnames (contrast) <- c("+wh-st", "+polar")
contrasts (internalH$sType) <- contrast
contrasts (internalH$sType)
```

```
##           +wh-st     +polar
## GQ           0.0  0.6666667
## PartQ        0.5 -0.3333333
## statement   -0.5 -0.3333333
```

We build the model where yInternal is predicted by emotion, sentence type, group, and all possible interactions, as well as random intercepts and slopes for emotion and group by sentence, and for emotion and sentence type by speaker.

Because models with 5 or less levels of a random factor often result in singular fits, we use a prior over the covarience matrix of random effects using the *blme* package.

```
library(blme) #bayesian model with covert prior for covariance matrix
library(optimx) #optimizer

modelIntbH<-blmer(x ~ emotion * sType + (emotion|sentence) + (emotion*sType|speaker_id), data=internalH, control = lmerControl(optimizer ='optimx', optCtrl=list(method='nlminb'))) #
summary(modelIntbH)
```

```
## Cov prior  : sentence ~ wishart(df = 5.5, scale = Inf, posterior.scale = cov, common.scale = TRUE)
##            : speaker_id ~ wishart(df = 11.5, scale = Inf, posterior.scale = cov, common.scale = TRUE)
## Prior dev  : 19.8358
## 
## Linear mixed model fit by REML ['blmerMod']
## Formula: x ~ emotion * sType + (emotion | sentence) + (emotion * sType |  
##     speaker_id)
##    Data: internalH
## Control: lmerControl(optimizer = "optimx", optCtrl = list(method = "nlminb"))
## 
## REML criterion at convergence: 1543.9
## 
## Scaled residuals: 
##     Min      1Q  Median      3Q     Max 
## -3.2694 -0.6015  0.0102  0.5709  4.0581 
## 
## Random effects:
##  Groups     Name                              Variance Std.Dev. Corr       
##  sentence   (Intercept)                        0.4425  0.6652              
##             emotion+neutral-anger              0.8612  0.9280   0.41       
##             emotion+surprise                   0.5380  0.7335   0.43  0.44 
##  speaker_id (Intercept)                       11.9918  3.4629              
##             emotion+neutral-anger             17.3515  4.1655    0.60      
##             emotion+surprise                   8.3325  2.8866    0.82  0.90
##             sType+wh-st                        2.6910  1.6404   -0.22 -0.49
##             sType+polar                        0.3478  0.5898   -0.14  0.20
##             emotion+neutral-anger:sType+wh-st  2.7221  1.6499   -0.53  0.02
##             emotion+surprise:sType+wh-st       8.3645  2.8921   -0.65 -0.29
##             emotion+neutral-anger:sType+polar 25.9404  5.0932    0.37  0.38
##             emotion+surprise:sType+polar       8.2291  2.8686   -0.86 -0.68
##  Residual                                      3.6080  1.8995              
##                                     
##                                     
##                                     
##                                     
##                                     
##                                     
##                                     
##  -0.58                              
##   0.19 -0.59                        
##  -0.22 -0.08  0.24                  
##  -0.61  0.62 -0.23  0.40            
##   0.59 -0.89  0.52 -0.10 -0.79      
##  -0.74  0.01  0.24  0.37  0.31 -0.06
##                                     
## Number of obs: 356, groups:  sentence, 10; speaker_id, 4
## 
## Fixed effects:
##                                   Estimate Std. Error t value
## (Intercept)                        26.6844     1.7471  15.274
## emotion+neutral-anger               4.7318     2.1176   2.234
## emotion+surprise                    3.8483     1.4775   2.605
## sType+wh-st                        -0.3060     0.8562  -0.357
## sType+polar                         3.1935     0.3647   8.757
## emotion+neutral-anger:sType+wh-st  -0.6663     1.0205  -0.653
## emotion+surprise:sType+wh-st       -3.0268     1.5376  -1.968
## emotion+neutral-anger:sType+polar   2.0179     2.5997   0.776
## emotion+surprise:sType+polar        0.1885     1.5056   0.125
## 
## Correlation of Fixed Effects:
##             (Intr) emtn+- emtn+s sTyp+- sTyp+p e+-:T+- e+:T+- em+-:T+
## emtn+ntrl-n  0.593                                                   
## emotn+srprs  0.803  0.871                                            
## sType+wh-st -0.213 -0.458 -0.544                                     
## sType+polar -0.109  0.161  0.151 -0.457                              
## emtn+n-:T+- -0.427  0.014 -0.171 -0.064  0.155                       
## emtn+sr:T+- -0.610 -0.268 -0.558  0.557 -0.174  0.302                
## emtn+nt-:T+  0.362  0.366  0.566 -0.835  0.410 -0.081  -0.731        
## emtn+srp:T+ -0.809 -0.638 -0.688  0.007  0.187  0.288   0.281 -0.055
```

```
library("car")
Anova(modelIntbH)
```

```
## Analysis of Deviance Table (Type II Wald chisquare tests)
## 
## Response: x
##                  Chisq Df Pr(>Chisq)    
## emotion        22.7416  2  1.153e-05 ***
## sType         103.1935  2  < 2.2e-16 ***
## emotion:sType   5.7283  4     0.2204    
## ---
## Signif. codes:  0 '***' 0.001 '**' 0.01 '*' 0.05 '.' 0.1 ' ' 1
```

We still observe the significant effects of both emotions and of polar questions, but not wh-questions.

We select the hearing signers only, external eyebrows.

```
externalH<-subset(external, group=="hearing")
```

We need to also need to inspect the values of yExternal to see if there are any unreasonable outliers. We do not see anything completely out of the ordinary here, so we can model it.

```
hist(externalH$x)
```

We set up orthogonal contrasts for our factors: we compare neutral to anger, and surprise to the average of neutral and anger, and we compare wh-questions to statements, and their average to polar questions.

```
contrast <- cbind (c(-1/2, 1/2, 0), c(-1/3, -1/3, 2/3))   # ang neut surpr
colnames (contrast) <- c("+neutral-anger", "+surprise")
contrasts (externalH$emotion) <- contrast
contrasts (externalH$emotion)
```

```
##         +neutral-anger  +surprise
## anger             -0.5 -0.3333333
## neutral            0.5 -0.3333333
## sur                0.0  0.6666667
```

```
contrast <- cbind (c(0, 1/2, -1/2), c(2/3, -1/3, -1/3))   # gq partQ statement
colnames (contrast) <- c("+wh-st", "+polar")
contrasts (externalH$sType) <- contrast
contrasts (externalH$sType)
```

```
##           +wh-st     +polar
## GQ           0.0  0.6666667
## PartQ        0.5 -0.3333333
## statement   -0.5 -0.3333333
```

We build the model where yExternal is predicted by emotion, sentence type, group, and all possible interactions, as well as random intercepts and slopes for emotion and group by sentence, and for emotion and sentence type by speaker.

Because models with 5 or less levels of a random factor often result in singular fits, we use a prior over the covarience matrix of random effects using the *blme* package.

```
library(blme) #bayesian model with covert prior for covariance matrix
library(optimx) #optimizer

modelExtbH<-blmer(x ~ emotion * sType + (emotion|sentence) + (emotion*sType|speaker_id), data=externalH, control = lmerControl(optimizer ='optimx', optCtrl=list(method='nlminb'))) #
summary(modelExtbH)
```

```
## Cov prior  : sentence ~ wishart(df = 5.5, scale = Inf, posterior.scale = cov, common.scale = TRUE)
##            : speaker_id ~ wishart(df = 11.5, scale = Inf, posterior.scale = cov, common.scale = TRUE)
## Prior dev  : 24.8167
## 
## Linear mixed model fit by REML ['blmerMod']
## Formula: x ~ emotion * sType + (emotion | sentence) + (emotion * sType |  
##     speaker_id)
##    Data: externalH
## Control: lmerControl(optimizer = "optimx", optCtrl = list(method = "nlminb"))
## 
## REML criterion at convergence: 1563.6
## 
## Scaled residuals: 
##     Min      1Q  Median      3Q     Max 
## -3.3719 -0.5755  0.0084  0.5100  3.2914 
## 
## Random effects:
##  Groups     Name                              Variance Std.Dev. Corr       
##  sentence   (Intercept)                        0.7550  0.8689              
##             emotion+neutral-anger              0.2202  0.4693   0.40       
##             emotion+surprise                   0.5829  0.7635   0.44  0.18 
##  speaker_id (Intercept)                        0.8762  0.9360              
##             emotion+neutral-anger             11.5150  3.3934   -0.68      
##             emotion+surprise                   3.2970  1.8158   -0.10  0.57
##             sType+wh-st                        3.9448  1.9861    0.87 -0.65
##             sType+polar                        2.0991  1.4488   -0.10 -0.10
##             emotion+neutral-anger:sType+wh-st  3.7951  1.9481   -0.20  0.61
##             emotion+surprise:sType+wh-st       1.7779  1.3334    0.14  0.13
##             emotion+neutral-anger:sType+polar 17.2516  4.1535   -0.46  0.02
##             emotion+surprise:sType+polar       7.8836  2.8078   -0.31 -0.37
##  Residual                                      3.8636  1.9656              
##                                     
##                                     
##                                     
##                                     
##                                     
##                                     
##                                     
##  -0.36                              
##   0.42 -0.42                        
##   0.31 -0.09 -0.43                  
##  -0.08  0.29 -0.52  0.33            
##   0.19 -0.67  0.81 -0.43 -0.55      
##  -0.40 -0.35  0.49 -0.59 -0.44  0.72
##                                     
## Number of obs: 357, groups:  sentence, 10; speaker_id, 4
## 
## Fixed effects:
##                                   Estimate Std. Error t value
## (Intercept)                        64.1969     0.5526 116.170
## emotion+neutral-anger               1.3269     1.7221   0.771
## emotion+surprise                    2.3941     0.9652   2.480
## sType+wh-st                        -0.8237     1.0251  -0.803
## sType+polar                         3.6328     0.7575   4.796
## emotion+neutral-anger:sType+wh-st  -0.8177     1.1555  -0.708
## emotion+surprise:sType+wh-st       -3.6104     0.8585  -4.206
## emotion+neutral-anger:sType+polar   1.7855     2.1460   0.832
## emotion+surprise:sType+polar        1.5691     1.4807   1.060
## 
## Correlation of Fixed Effects:
##             (Intr) emtn+- emtn+s sTyp+- sTyp+p e+-:T+- e+:T+- em+-:T+
## emtn+ntrl-n -0.551                                                   
## emotn+srprs -0.026  0.537                                            
## sType+wh-st  0.715 -0.620 -0.324                                     
## sType+polar -0.085 -0.094  0.380 -0.390                              
## emtn+n-:T+- -0.140  0.510  0.243 -0.074 -0.345                       
## emtn+sr:T+-  0.094  0.096 -0.060  0.220 -0.384  0.218                
## emtn+nt-:T+ -0.377  0.016  0.174 -0.625  0.753 -0.349  -0.410        
## emtn+srp:T+ -0.249 -0.346 -0.356 -0.322  0.444 -0.472  -0.327  0.660
```

```
library("car")
Anova(modelExtbH)
```

```
## Analysis of Deviance Table (Type II Wald chisquare tests)
## 
## Response: x
##                Chisq Df Pr(>Chisq)    
## emotion       12.422  2  0.0020069 ** 
## sType         38.302  2  4.817e-09 ***
## emotion:sType 18.759  4  0.0008764 ***
## ---
## Signif. codes:  0 '***' 0.001 '**' 0.01 '*' 0.05 '.' 0.1 ' ' 1
```

Here we still see the expected effects of surprise, and the effect of polar questions. The interaction between emotion and sentence type is also significant due to the interaction between surprise and wh-questions.

## Right-left asymmetry

We noticed (and also a reviwer at PLOS ONE pointed out) that the right and left eyebrows behave differently. To assess the asymmetry we first create a new measure, namely the difference between left and right internal and external eyebrow position.

We retrace the steps used for the average eyebrow positions.

```
keypoints3$asymInternal<-(keypoints3$rd21-keypoints3$rd22)
keypoints3$asymExternal<-(keypoints3$rd18-keypoints3$rd25)

keypointsD3$asymInternal<-(keypointsD3$rd21-keypointsD3$rd22)
keypointsD3$asymExternal<-(keypointsD3$rd18-keypointsD3$rd25)

hist(keypoints3$asymInternal) #remove outliers
```

```
keypoints3$asymInternal[keypoints3$asymInternal>50|keypoints3$asymInternal<(-50)]<-NA
hist(keypoints3$asymInternal) #remove outliers
```

```
hist(keypoints3$asymExternal)
```

```
keypoints3$asymExternal[keypoints3$asymExternal>50|keypoints3$asymExternal<(-50)]<-NA

hist(keypointsD3$asymInternal)
```

```
hist(keypointsD3$asymExternal)
```

```
keypoints.asym<-data.frame(keypoints3$asymInternal, keypoints3$asymExternal, keypoints3$sentenceID, keypoints3$signerID, keypoints3$sType, keypoints3$emotion)

keypoints.asym$group<-"hearing"
colnames(keypoints.asym)<-c("asymInternal", "asymExternal", "sentence", "signer", "sType", "emotion", "group")

keypoints.2<-data.frame(keypointsD3$asymInternal, keypointsD3$asymExternal, keypointsD3$sentence, keypointsD3$speaker_id, keypointsD3$sType, keypointsD3$emotion)

keypoints.2$group<-"deaf"

colnames(keypoints.2)<-c("asymInternal", "asymExternal", "sentence", "signer", "sType", "emotion", "group")


keypoints.asym<-rbind(keypoints.asym, keypoints.2)

#the levels for sentences are not the same in the two datasets, so we change this
keypoints.all$sentence[keypoints.all$sentence=="dev_upala"]<-"devupala"
keypoints.all$sentence[keypoints.all$sentence=="dom_post"]<-"dom.postroili"
keypoints.all$sentence[keypoints.all$sentence=="mal_chit"]<-"mal.chit"
keypoints.all$sentence[keypoints.all$sentence=="mama_ust"]<-"mama.ustala"
keypoints.all$sentence[keypoints.all$sentence=="okno_razb"]<-"okno.razb"
keypoints.all$sentence[keypoints.all$sentence=="papa_beg"]<-"papa.bezhit"
keypoints.all$sentence[keypoints.all$sentence=="reb_tanc"]<-"reb.tanc"
keypoints.all$sentence[keypoints.all$sentence=="sob_est"]<-"sobaka.est"
keypoints.all$sentence[keypoints.all$sentence=="tel_slom"]<-"tel.slom"
keypoints.all$sentence[keypoints.all$sentence=="uchit_smeh"]<-"uch.smeh"
keypoints.all$sentence<-droplevels(keypoints.all$sentence)
```

We then aggregate the data, as we did for the analysis of the average between the two eyebrows, and we model the asymmetry as predicted by group, sentence type, emotion, as well as the random factors of sentence and signer. First for the asymmetry in the internal positions:

```
asymInternal<-aggregate(keypoints.asym$asymInternal, by=list(keypoints.asym$signer, keypoints.asym$sentence, keypoints.asym$sType, keypoints.asym$emotion, keypoints.asym$group), mean)
colnames(asymInternal)<-c('speaker_id', 'sentence', 'sType', 'emotion', 'group', 'asymInt')

hist(asymInternal$asymInt) #no obvious outliers
```

```
contrast <- cbind (c(-1/2, 1/2, 0), c(-1/3, -1/3, 2/3))   # ang neut surpr
colnames (contrast) <- c("+neutral-anger", "+surprise")
contrasts (asymInternal$emotion) <- contrast
contrasts (asymInternal$emotion)
```

```
##         +neutral-anger  +surprise
## anger             -0.5 -0.3333333
## neutral            0.5 -0.3333333
## sur                0.0  0.6666667
```

```
contrast <- cbind (c(0, 1/2, -1/2), c(2/3, -1/3, -1/3))   # gq partQ statement
colnames (contrast) <- c("+wh-st", "+polar")
contrasts (asymInternal$sType) <- contrast
contrasts (asymInternal$sType)
```

```
##           +wh-st     +polar
## GQ           0.0  0.6666667
## PartQ        0.5 -0.3333333
## statement   -0.5 -0.3333333
```

```
asymInternal$group<-as.factor(asymInternal$group)


contrast <- cbind (c(1/2, -1/2)) #deaf, hearing
colnames (contrast) <- c("+deaf")
contrasts (asymInternal$group) <- contrast
contrasts(asymInternal$group)
```

```
##         +deaf
## deaf      0.5
## hearing  -0.5
```

```
library(blme) #bayesian model with covert prior for covariance matrix
library(optimx) #optimizer

modelIntAsym<-blmer(asymInt ~ emotion * sType * group + (emotion*group|sentence) + (emotion*sType|speaker_id), data=asymInternal, control = lmerControl(optimizer ='optimx', optCtrl=list(method='nlminb'))) 
summary(modelIntAsym)
```

```
## Cov prior  : sentence ~ wishart(df = 8.5, scale = Inf, posterior.scale = cov, common.scale = TRUE)
##            : speaker_id ~ wishart(df = 11.5, scale = Inf, posterior.scale = cov, common.scale = TRUE)
## Prior dev  : 45.8412
## 
## Linear mixed model fit by REML ['blmerMod']
## Formula: asymInt ~ emotion * sType * group + (emotion * group | sentence) +  
##     (emotion * sType | speaker_id)
##    Data: asymInternal
## Control: lmerControl(optimizer = "optimx", optCtrl = list(method = "nlminb"))
## 
## REML criterion at convergence: 2454.6
## 
## Scaled residuals: 
##     Min      1Q  Median      3Q     Max 
## -4.7527 -0.5426  0.0040  0.5128  5.3033 
## 
## Random effects:
##  Groups     Name                              Variance Std.Dev. Corr       
##  sentence   (Intercept)                       0.02740  0.1655              
##             emotion+neutral-anger             0.05953  0.2440    0.53      
##             emotion+surprise                  0.03857  0.1964    0.59  0.41
##             group+deaf                        0.10963  0.3311   -0.16 -0.11
##             emotion+neutral-anger:group+deaf  0.23810  0.4880   -0.11  0.19
##             emotion+surprise:group+deaf       0.15429  0.3928   -0.11  0.01
##  speaker_id (Intercept)                       1.52695  1.2357              
##             emotion+neutral-anger             0.77958  0.8829   -0.27      
##             emotion+surprise                  0.09957  0.3156   -0.25  0.70
##             sType+wh-st                       0.67722  0.8229   -0.11 -0.71
##             sType+polar                       0.29943  0.5472   -0.01 -0.19
##             emotion+neutral-anger:sType+wh-st 0.85167  0.9229    0.69  0.07
##             emotion+surprise:sType+wh-st      0.63760  0.7985    0.03  0.50
##             emotion+neutral-anger:sType+polar 0.80564  0.8976    0.14 -0.34
##             emotion+surprise:sType+polar      0.53070  0.7285    0.07  0.59
##  Residual                                     1.03242  1.0161              
##                                     
##                                     
##                                     
##                                     
##  -0.11                              
##   0.01  0.53                        
##  -0.15  0.59  0.41                  
##                                     
##                                     
##                                     
##  -0.37                              
##   0.26 -0.02                        
##  -0.03 -0.47  0.26                  
##   0.17 -0.14 -0.71 -0.04            
##  -0.19  0.12 -0.01 -0.28 -0.39      
##   0.29 -0.22 -0.67  0.00  0.86 -0.34
##                                     
## Number of obs: 803, groups:  sentence, 20; speaker_id, 9
## 
## Fixed effects:
##                                               Estimate Std. Error t value
## (Intercept)                                   0.243427   0.419317   0.581
## emotion+neutral-anger                         0.005455   0.318553   0.017
## emotion+surprise                             -0.472652   0.144659  -3.267
## sType+wh-st                                  -0.034157   0.289819  -0.118
## sType+polar                                  -0.047883   0.198904  -0.241
## group+deaf                                    0.630100   0.838633   0.751
## emotion+neutral-anger:sType+wh-st             0.119307   0.377727   0.316
## emotion+surprise:sType+wh-st                  0.021038   0.326889   0.064
## emotion+neutral-anger:sType+polar             0.299818   0.354790   0.845
## emotion+surprise:sType+polar                 -0.404202   0.293534  -1.377
## emotion+neutral-anger:group+deaf             -1.116900   0.637106  -1.753
## emotion+surprise:group+deaf                   0.169838   0.289317   0.587
## sType+wh-st:group+deaf                        0.800663   0.579639   1.381
## sType+polar:group+deaf                        0.870623   0.397808   2.189
## emotion+neutral-anger:sType+wh-st:group+deaf  0.325377   0.755454   0.431
## emotion+surprise:sType+wh-st:group+deaf       0.205654   0.653777   0.315
## emotion+neutral-anger:sType+polar:group+deaf  0.841254   0.709580   1.186
## emotion+surprise:sType+polar:group+deaf      -0.400603   0.587069  -0.682
```

```
## 
## Correlation matrix not shown by default, as p = 18 > 12.
## Use print(x, correlation=TRUE)  or
##     vcov(x)        if you need it
```

```
library("car")
Anova(modelIntAsym)
```

```
## Analysis of Deviance Table (Type II Wald chisquare tests)
## 
## Response: asymInt
##                       Chisq Df Pr(>Chisq)    
## emotion             13.9249  2  0.0009468 ***
## sType                0.1852  2  0.9115808    
## group                0.0558  1  0.8132051    
## emotion:sType        4.6454  4  0.3256529    
## emotion:group        6.3679  2  0.0414210 *  
## sType:group         11.3132  2  0.0034943 ** 
## emotion:sType:group  3.2562  4  0.5159078    
## ---
## Signif. codes:  0 '***' 0.001 '**' 0.01 '*' 0.05 '.' 0.1 ' ' 1
```

Significant effect of emotion (surprise), emotion-group interaction, and sentence type group interaction (GQ and deaf).

Now let’s do the same for the external eyebrow asymmetry:

```
asymExternal<-aggregate(keypoints.asym$asymExternal, by=list(keypoints.asym$signer, keypoints.asym$sentence, keypoints.asym$sType, keypoints.asym$emotion, keypoints.asym$group), mean)
colnames(asymExternal)<-c('speaker_id', 'sentence', 'sType', 'emotion', 'group', 'asymExt')

hist(asymExternal$asymExt) #no clear outliers
```

```
contrast <- cbind (c(-1/2, 1/2, 0), c(-1/3, -1/3, 2/3))   # ang neut surpr
colnames (contrast) <- c("+neutral-anger", "+surprise")
contrasts (asymExternal$emotion) <- contrast
contrasts (asymExternal$emotion)
```

```
##         +neutral-anger  +surprise
## anger             -0.5 -0.3333333
## neutral            0.5 -0.3333333
## sur                0.0  0.6666667
```

```
contrast <- cbind (c(0, 1/2, -1/2), c(2/3, -1/3, -1/3))   # gq partQ statement
colnames (contrast) <- c("+wh-st", "+polar")
contrasts (asymExternal$sType) <- contrast
contrasts (asymExternal$sType)
```

```
##           +wh-st     +polar
## GQ           0.0  0.6666667
## PartQ        0.5 -0.3333333
## statement   -0.5 -0.3333333
```

```
asymExternal$group<-as.factor(asymExternal$group)


contrast <- cbind (c(1/2, -1/2)) #deaf, hearing
colnames (contrast) <- c("+deaf")
contrasts (asymExternal$group) <- contrast
contrasts(asymExternal$group)
```

```
##         +deaf
## deaf      0.5
## hearing  -0.5
```

```
library(blme) #bayesian model with covert prior for covariance matrix
library(optimx) #optimizer

modelExtAsym<-blmer(asymExt ~ emotion * sType * group + (emotion*group|sentence) + (emotion*sType|speaker_id), data=asymExternal, control = lmerControl(optimizer ='optimx', optCtrl=list(method='nlminb'))) 
summary(modelExtAsym)
```

```
## Cov prior  : sentence ~ wishart(df = 8.5, scale = Inf, posterior.scale = cov, common.scale = TRUE)
##            : speaker_id ~ wishart(df = 11.5, scale = Inf, posterior.scale = cov, common.scale = TRUE)
## Prior dev  : 50.2408
## 
## Linear mixed model fit by REML ['blmerMod']
## Formula: asymExt ~ emotion * sType * group + (emotion * group | sentence) +  
##     (emotion * sType | speaker_id)
##    Data: asymExternal
## Control: lmerControl(optimizer = "optimx", optCtrl = list(method = "nlminb"))
## 
## REML criterion at convergence: 4731.7
## 
## Scaled residuals: 
##     Min      1Q  Median      3Q     Max 
## -4.4312 -0.5629  0.0103  0.5284  3.5412 
## 
## Random effects:
##  Groups     Name                              Variance Std.Dev. Corr       
##  sentence   (Intercept)                        0.7937  0.8909              
##             emotion+neutral-anger              0.8944  0.9457    0.43      
##             emotion+surprise                   1.2996  1.1400    0.66  0.08
##             group+deaf                         3.1746  1.7818   -0.32 -0.11
##             emotion+neutral-anger:group+deaf   3.5777  1.8915   -0.11  0.13
##             emotion+surprise:group+deaf        5.1981  2.2799   -0.27 -0.33
##  speaker_id (Intercept)                       12.6861  3.5618              
##             emotion+neutral-anger             10.7827  3.2837   -0.54      
##             emotion+surprise                   1.7554  1.3249    0.62  0.12
##             sType+wh-st                       10.6787  3.2678    0.26 -0.89
##             sType+polar                        1.3834  1.1762   -0.20  0.60
##             emotion+neutral-anger:sType+wh-st  7.6697  2.7694   -0.12  0.57
##             emotion+surprise:sType+wh-st       9.7380  3.1206   -0.05 -0.61
##             emotion+neutral-anger:sType+polar  5.0995  2.2582    0.45 -0.61
##             emotion+surprise:sType+polar      19.8956  4.4604   -0.23 -0.07
##  Residual                                     18.9724  4.3557              
##                                     
##                                     
##                                     
##                                     
##  -0.27                              
##  -0.33  0.43                        
##  -0.12  0.66  0.08                  
##                                     
##                                     
##                                     
##  -0.37                              
##   0.39 -0.66                        
##   0.28 -0.69  0.44                  
##  -0.58  0.75 -0.72 -0.62            
##   0.14  0.58 -0.21 -0.58  0.32      
##  -0.49  0.17 -0.61 -0.09  0.55 -0.25
##                                     
## Number of obs: 804, groups:  sentence, 20; speaker_id, 9
## 
## Fixed effects:
##                                              Estimate Std. Error t value
## (Intercept)                                   0.55244    1.23712   0.447
## emotion+neutral-anger                         3.13306    1.20243   2.606
## emotion+surprise                             -0.07166    0.65967  -0.109
## sType+wh-st                                  -0.19842    1.15952  -0.171
## sType+polar                                   0.08127    0.51334   0.158
## group+deaf                                    2.27255    2.47424   0.918
## emotion+neutral-anger:sType+wh-st             0.97290    1.31179   0.742
## emotion+surprise:sType+wh-st                 -1.01078    1.31919  -0.766
## emotion+neutral-anger:sType+polar             0.55695    1.10468   0.504
## emotion+surprise:sType+polar                 -1.29155    1.65054  -0.783
## emotion+neutral-anger:group+deaf             -5.01704    2.40486  -2.086
## emotion+surprise:group+deaf                   1.38630    1.31934   1.051
## sType+wh-st:group+deaf                        3.46250    2.31904   1.493
## sType+polar:group+deaf                        2.48226    1.02669   2.418
## emotion+neutral-anger:sType+wh-st:group+deaf -0.59911    2.62359  -0.228
## emotion+surprise:sType+wh-st:group+deaf       2.92350    2.63839   1.108
## emotion+neutral-anger:sType+polar:group+deaf  4.91640    2.20937   2.225
## emotion+surprise:sType+polar:group+deaf      -1.94555    3.30108  -0.589
```

```
## 
## Correlation matrix not shown by default, as p = 18 > 12.
## Use print(x, correlation=TRUE)  or
##     vcov(x)        if you need it
```

```
library("car")
Anova(modelExtAsym)
```

```
## Analysis of Deviance Table (Type II Wald chisquare tests)
## 
## Response: asymExt
##                       Chisq Df Pr(>Chisq)   
## emotion              8.2817  2   0.015910 * 
## sType                0.1474  2   0.928973   
## group                0.2423  1   0.622519   
## emotion:sType        1.9977  4   0.736182   
## emotion:group        2.6764  2   0.262314   
## sType:group         13.4361  2   0.001209 **
## emotion:sType:group  6.3775  4   0.172677   
## ---
## Signif. codes:  0 '***' 0.001 '**' 0.01 '*' 0.05 '.' 0.1 ' ' 1
```

Significant effect of emotion (anger) and type-group interaction (polar question and deaf).

We do not know how to interpret asymmetry in general, and specifically its correlation with some of the conditions.
