# Supplementary material for: Eyebrow position in grammatical and emotional expressions in Kazakh-Russian Sign Language: A quantitative study
Source: PLoS One. 2020 Jun 2;15(6):e0233731. doi: 10.1371/journal.pone.0233731 (PMC7266324; doi:10.1371/journal.pone.0233731)
Supplement: S2 Appendix — This Appendix contains the statistical analysis (in RMarkdown format and exported to html) of the validation experiment and the full data table used in the analysis. (ZIP) [file pone.0233731.s002.zip › validation files/validation-analysis.html]

Validation eyebrows


Code 

- Show All Code
- Hide All Code

# Validation eyebrows

#### Vadim Kimmelman

#### 12/13/2019

---

In this script, we assess the validity of the emotional facial expressions by analysing the results of a validation experiment. 81 videos from the analysed data were shown to 5 deaf signers of KRSL. They were asked to determine emotions (with four options: surprise, anger, neutral, other). The results are coded in a table with the responder’s codes, their reponses, and the label for the video that was used.

The only file necessary to run the script is “validation results.txt”. It contains 405 lines (81 sentences by 5 participants).

## Importing and preparing the data

First we import the data, and also create the columns with intended emotions using the video labels. We also create a column for a match between the true and false emotions. In some cases (23 cases), the participant selected more than one option, which we can treat either as correct or incorrect. To treat those cases, we create a column “double” where such cases are marked.

```
validation.results <- read.delim("validation results.txt", na.strings="")

#let's creat columns for true emotions
validation.results$Tneutral<-NA
validation.results$Tsurprised<-NA
validation.results$Tangry<-NA

#now we fill in the columns for emotions if this emotion is mentioned in X
validation.results$Tneutral[grepl("neut", as.character(validation.results$X))]<-1
validation.results$Tsurprised[grepl("sur", as.character(validation.results$X))]<-1
validation.results$Tangry[grepl("anger", as.character(validation.results$X))]<-1

#now let's creat the match column and fill it in 
validation.results$match<-0
validation.results$match[validation.results$Tneutral==validation.results$neutral]<-1
validation.results$match[validation.results$Tangry==validation.results$angry]<-1
validation.results$match[validation.results$Tsurprised==validation.results$surprised]<-1

#this way matches are counted also when someone selected two emotions
#let's mark the rows where this is the case for a more conservative measure
validation.results$double<-0
validation.results$double[validation.results$surprised==1&validation.results$neutral==1|validation.results$surprised==1&validation.results$angry==1|validation.results$angry==1&validation.results$neutral==1]<-1
sum(validation.results$double) #23 cases
```

```
## [1] 23
```

## Looking at the results

First, we look at the results in general descriptive terms, in total, as well as per emotion and per signer.

```
#proportion of overall correct
sum(validation.results$match)/length(validation.results$match) #67%
```

```
## [1] 0.6716049
```

```
#or if we are more conservative and discount the doubly marked emotions
(sum(validation.results$match)-23)/length(validation.results$match) #61%
```

```
## [1] 0.6148148
```

```
#chance correct would be 0.33

#proportion of correct per emotion
sum(na.omit(validation.results$match[validation.results$Tneutral==1]))/length(na.omit(validation.results$match[validation.results$Tneutral==1])) #62%
```

```
## [1] 0.6266667
```

```
sum(na.omit(validation.results$match[validation.results$Tsurprised==1]))/length(na.omit(validation.results$match[validation.results$Tsurprised==1])) #68%
```

```
## [1] 0.6888889
```

```
sum(na.omit(validation.results$match[validation.results$Tangry==1]))/length(na.omit(validation.results$match[validation.results$Tangry==1])) #70%
```

```
## [1] 0.7083333
```

```
#proportion of correct per signer
table<-aggregate(validation.results$match, by=list(validation.results$subject), FUN=sum)
table$proportion<-table$x/81
table
```

```
##   Group.1  x proportion
## 1      s1 62  0.7654321
## 2      s2 57  0.7037037
## 3      s3 54  0.6666667
## 4      s4 51  0.6296296
## 5      s5 48  0.5925926
```

```
#some are better then others, but all are well above chance level
```

The results are an average of 67% agreement (or 61% if we treat the doubly marked emotions as errors). Neutral emotion is slighly more diffcult to identify than surprise or anger. There is some variation between participants, from 59 to 76% of correct responses.

## Assessing relative to chance level

Because there is a possiblity of chance correct answers due to the limited number of options, we have to account for chance agreement.

First, we calculate Cohen’s kappa per person (agreement between each person and intended emotions), and also Light’s kappa for agreement (agreement between the 5 participants). To do so we also need to reshape the table.

```
#first we need to create categories for the answers and for the correct emotions in stimuli
validation.results$judgment<-"NA"
#the &validation.results$double==0 marks the cases where two judgments were made as incorrect
validation.results$judgment[validation.results$neutral==1&validation.results$double==0]<-"neutral"
validation.results$judgment[validation.results$surprised==1&validation.results$double==0]<-"surprised"
validation.results$judgment[validation.results$angry==1&validation.results$double==0]<-"angry"

validation.results$stimulus<-"NA"
validation.results$stimulus[validation.results$Tneutral==1]<-"neutral"
validation.results$stimulus[validation.results$Tsurprised==1]<-"surprised"
validation.results$stimulus[validation.results$Tangry==1]<-"angry"


library("psych")
cohen.kappa(cbind(validation.results$judgment[validation.results$subject=="s1"],validation.results$stimulus[validation.results$subject=="s1"])) #34
```

```
## Call: cohen.kappa1(x = x, w = w, n.obs = n.obs, alpha = alpha, levels = levels)
## 
## Cohen Kappa and Weighted Kappa correlation coefficients and confidence boundaries 
##                  lower estimate upper
## unweighted kappa  0.22     0.34  0.47
## weighted kappa    0.27     0.50  0.73
## 
##  Number of subjects = 81
```

```
cohen.kappa(cbind(validation.results$judgment[validation.results$subject=="s2"],validation.results$stimulus[validation.results$subject=="s2"])) #56
```

```
## Call: cohen.kappa1(x = x, w = w, n.obs = n.obs, alpha = alpha, levels = levels)
## 
## Cohen Kappa and Weighted Kappa correlation coefficients and confidence boundaries 
##                  lower estimate upper
## unweighted kappa  0.42     0.56   0.7
## weighted kappa    0.70     0.70   0.7
## 
##  Number of subjects = 81
```

```
cohen.kappa(cbind(validation.results$judgment[validation.results$subject=="s3"],validation.results$stimulus[validation.results$subject=="s3"])) #51
```

```
## Call: cohen.kappa1(x = x, w = w, n.obs = n.obs, alpha = alpha, levels = levels)
## 
## Cohen Kappa and Weighted Kappa correlation coefficients and confidence boundaries 
##                  lower estimate upper
## unweighted kappa  0.36     0.51  0.66
## weighted kappa    0.62     0.68  0.74
## 
##  Number of subjects = 81
```

```
cohen.kappa(cbind(validation.results$judgment[validation.results$subject=="s4"],validation.results$stimulus[validation.results$subject=="s4"])) #49
```

```
## Call: cohen.kappa1(x = x, w = w, n.obs = n.obs, alpha = alpha, levels = levels)
## 
## Cohen Kappa and Weighted Kappa correlation coefficients and confidence boundaries 
##                  lower estimate upper
## unweighted kappa  0.36     0.49  0.63
## weighted kappa    0.62     0.71  0.80
## 
##  Number of subjects = 81
```

```
cohen.kappa(cbind(validation.results$judgment[validation.results$subject=="s5"],validation.results$stimulus[validation.results$subject=="s5"])) #39
```

```
## Call: cohen.kappa1(x = x, w = w, n.obs = n.obs, alpha = alpha, levels = levels)
## 
## Cohen Kappa and Weighted Kappa correlation coefficients and confidence boundaries 
##                   lower estimate upper
## unweighted kappa  0.229     0.39  0.54
## weighted kappa   -0.037     0.30  0.63
## 
##  Number of subjects = 81
```

```
#this is fair to moderate agreement

#let's also do Light's kappa for a single measurement of agreement between the raters
ratings<-cbind(validation.results$judgment[validation.results$subject=="s1"], validation.results$judgment[validation.results$subject=="s2"], validation.results$judgment[validation.results$subject=="s3"], validation.results$judgment[validation.results$subject=="s4"], validation.results$judgment[validation.results$subject=="s5"])


library("irr")
```

```
## Loading required package: lpSolve
```

```
kappam.light(ratings) #0.398, p=0.006
```

```
##  Light's Kappa for m Raters
## 
##  Subjects = 81 
##    Raters = 5 
##     Kappa = 0.398 
## 
##         z = 2.77 
##   p-value = 0.00564
```

We find that Cohen’s kappa is from 0.39 to 0.56 depending on the subject, but it is significantly higher than 0 for all of them. This is fair to moderate agreement. Light’s kappa for overall agreement is 0.398, p=0.006.

Note that the kappas’ calculations were based on the assumption that there are 3 cateogries, not 4 as was in fact the case, so they are more conservative.

## A more common way of correcting for chance agreement

In the literature on emotion perception, a slighly different formula is used to correct for chance agreement:

“We used the standard correction formula, (proportion correct - (1/number of choices))/ (1 - (1/number of choices)), (Nunnally & Bernstein, 1994). Conceptually, this means that we subtract the portion of the accuracy that is due to chance, and then we scale the remainder of the score by the new total score possible” (Elfenbein & Ambday 2002: 208)

In order to be able to compare our results with reported research, we also used this formula.

```
#correction for chance agreement
((sum(validation.results$match)-23)/length(validation.results$match) - (1/3))/(1-1/3) #42%
```

```
## [1] 0.4222222
```

```
#however, the real number of categories is 4, because there was also the option "other"
((sum(validation.results$match)-23)/length(validation.results$match) - (1/4))/(1-1/4) #48%
```

```
## [1] 0.4864198
```

We can calculate it in two ways: as if the test has 3 categories, or as if it has 4 categories (because the option “other” was also given). The first gives us the 0.42 result, and the second the 0.48.

## Is recognition worse for grammatically marked sentences?

Let’s see whether emotions in questions are more difficult to recognize than in statements. In order to do that, we create a new column with sentence type information, and compare agreement per sentence type (considering double marking as disagreement).

```
#now we fill in the columns for emotions if this emotion is mentioned in X
validation.results$sentence<-NA
validation.results$sentence[grepl("utv", as.character(validation.results$X))]<-"statement"
validation.results$sentence[grepl("gen_q", as.character(validation.results$X))]<-"polar"
validation.results$sentence[grepl("part_q", as.character(validation.results$X))]<-"wh"

#proportion of correct per emotion
sum(na.omit(validation.results$match[validation.results$sentence=="statement"&validation.results$double==0]))/length(na.omit(validation.results$match[validation.results$sentence=="statement"])) #73%
```

```
## [1] 0.7259259
```

```
sum(na.omit(validation.results$match[validation.results$sentence=="polar"&validation.results$double==0]))/length(na.omit(validation.results$match[validation.results$sentence=="polar"])) #58%
```

```
## [1] 0.5769231
```

```
sum(na.omit(validation.results$match[validation.results$sentence=="wh"&validation.results$double==0]))/length(na.omit(validation.results$match[validation.results$sentence=="wh"])) #56%
```

```
## [1] 0.5571429
```
