# Supplementary material for: Eyebrow position in grammatical and emotional expressions in Kazakh-Russian Sign Language: A quantitative study
Source: PLoS One. 2020 Jun 2;15(6):e0233731. doi: 10.1371/journal.pone.0233731 (PMC7266324; doi:10.1371/journal.pone.0233731)
Supplement: S1 Table — (PDF) [file pone.0233731.s003.pdf]

S1 Table. S1 Table contains the sentences used in this study.

S1 **Table. Sentences used in the study (translated to English).**

	**Statement**	**Polar question**	**Wh-question**
1	The girl fell down.	Did the girl fall down?	Where did the girl fall down?
2	The boy is reading.	Is the boy reading?	What is the boy reading?
3	The TV is broken.	Is the TV broken?	Why is the TV broken?
4	The window shattered.	Did the window shatter?	Why did the window shatter?
5	The teacher is laughing.	Is the teacher laughing?	Why is the teacher laughing?
6	Mother is tired.	Is mother tired?	When is mother tired?
7	The house was built.	Was the house built?	When was the house built?
8	Father is running.	Is father running?	Where is father running?
9	The dog is eating.	IS the dog eating.	What is the dog eating?
10	The child is dancing.	Is the child dancing?	Where is the child dancing?
