# Supplementary material for: Eyebrow position in grammatical and emotional expressions in Kazakh-Russian Sign Language: A quantitative study
Source: PLoS One. 2020 Jun 2;15(6):e0233731. doi: 10.1371/journal.pone.0233731 (PMC7266324; doi:10.1371/journal.pone.0233731)
Supplement: Attachment — Submitted filename: eyebrows PLOS ONE reply to reviews.docx [file pone.0233731.s006.docx]

*We are grateful to the reviewers and the editor for their detailed feedback. Below we address the points raised by the reviewers and describe the changes we implemented. Reviewer 2 did not provide any suggestions for changes, so we do not discuss their review below. All the implemented changes are highlighted with blue text color in the marked up copy of the manuscript.*

*Concerning the S1 Video (and also an additional S2 Video, which contains the same material but with OpenPose keypoints overimposed), we have obtained permission from the participant and archived the consent form. The relevant line is added to the Methodology section.*

*We have also checked that the manuscript meets the PLOS style requirements.*

*Before discussing the reviews, we need to mention a change that we implemented that the reviewers did not ask for. We changed the method of calculating eyebrow height. In the first version, we used the difference of the y-coordinates for the eyebrow keypoints and the top of the nose. This measure could potentially be affected by head and body leans. To address this, we now calculate the distance between pairs of keypoints using both x and y coordinates (the usual distance formula calculation). Note that the results do not substantially change: all the significant effects are in the same direction, but we do find one additional significant interaction, which is also discussed.*

Reviewer #1: It is great to see the field for automatic sign language analysis advancing, in particular for the painstaking analyses of facial expressions! My comments below are aimed at polishing this clearly structured and relevant paper.

For ease of reading consider the gender-neutral use of the pronoun “they” as an alternative to s/he.

*Done*

Lines 77-93 Please note that de Vos et al.’s methodology (specifically sentence types) is not summarized accurately. That is to say, only a subset of the data (polar and content questions) were included in the analysis.

*Corrected.*

In line 135 the authors claim that “RSL and KRSL are closely related and very similar lexically”. For those unfamiliar with the political history, could you please say something more about when these languages may have started to diverge, and how likely it is that the two communities are still in regular contact.

*We added the basic historical information, but we are not aware of any research discussing these issues.*

Line 147-148 Kazakhstan’s government provides 60 free hours of sign language interpreter service support for each deaf person, which could be spent on medical, legal, or other communication needs.

>Are these hours given each year?

*Yes, the phrase “per year” now added to the sentence.*

Line 200 Did you consider doing a debriefing/post-hoc interview? Why not?

*We did not conduct an extended debriefing because the purpose of the study was disclosed to the participants in advance. The participants did however get the opportunity to ask questions and/or provide feedback. This information is now added in the Design of the study section.*

Line 291 What was the frame rate?

*30 fps, information now added to the sentence*

Line 357-364 With respect to the pixel analysis, I would like to know whether you have evidence that these are actually perceivable differences for signers themselves?

*The only evidence we have is the validation study described above. We have shown that the signers are able to recognize emotions above the chance level, also in combination with question marking. However, it is impossible to be sure that this recognition is due to perception of eyebrow position alone, because the signers might (and very likely are) using other non-manual clues when recognizing emotions and sentence types.*

Line 464-480 Do you think that the interpreters/CODAs may also have reacted differently because they were presented with different stimuli? (written text as opposed to signed videos).

*This is a possibility, we now added a discussion of it to the relevant section.*

Since you have set it up so nicely, I think it would be worthwhile revisiting de Vos et al.’s Affect over Grammar hypothesis in the conclusion section.

*We have slightly reformulated the discussion of this hypothesis in the Discussion section, and we feel that we do not need to mention it again in the conclusions.*

Reviewer #3: The work reported in this manuscript is well motivated, it addresses an important question regarding interaction of affective and linguistic facial expressions during sign language production. Unlike two similar previous studies (De Vos et al. (2009) and Weast (2011), the authors of this manuscript tracked and analyzed subjects’ eyebrow movements during sign production with OpenPose software, which allowed for more precise quantitative and statistical comparisons. Overall, I found this paper to be clearly written. The motivation for the study is well outlined and the results are clearly interpreted.

Only a few minor points:

1. The number of subjects (9) in the study is very small to make some generalizations based on the statistical findings presented in the manuscript; however, the study design and methodology are solid. Given that the authors have explicitly acknowledged some important caveats in their conclusion, the editor may want to consider the manuscript seriously despite a small sample size.

*We acknowledged the exploratory nature of this research in the manuscript, so it seems that this comment does not require revisions of the manuscript.*

2. Pg 8, Line 328. The number of measurements should be 810. If some measurements were excluded then the authors should state reasons for those exclusions.

*This is correct, the five missing data points are sentences for which OpenPose gave low confidence of detection of keypoints (below 0.7) for all the analyzed eyebrow keypoints in the investigated interval (between 20 and 80% of the video duration). We added this information to the paper in the Statistical Analysis section, and also to the Rmd file with the full description of the analysis.*

3. The authors should include an image in the manuscript (or a video in the supplemental materials) showing how well the OpenPose software tracks the eyebrow movements.

*We included a video in supplemental materials (S2 Video) illustrating OpenPose keypoints overlaid over the same video used in S1 Video.*

4. For all figures, align the emotion labels to the eyebrows. Some labels were off to one side or another.

*We have decided that the figures are more misleading than helpful, and changed them to a more traditional representation of the variables investigated (boxplots).*

5. Did authors analyze left / right asymmetry in the eyebrow positions, particularly for the polar question across the groups? The figure 2 (Hearing signers) seems to show a right-toward asymmetry in the eyebrow position for polar questions, whereas figure 3 (Deaf signers) shows the asymmetry in the opposite direction.

*While we noticed it as well, we had not conducted any analysis prior to manuscript submission. However, we now have conducted a statistical analysis Supplement, and we mention the issue in a footnote in the paper. There is indeed some asymmetry between the eyebrows, and it is influenced by some of the factors considered. We do not however have a good explanation for this finding, so this is left for future research.*

6. Since all signers were instructed to repeat signing a combination of sentence type and emotion 10 times for 9 different combinations. Were those combinations counterbalanced across signers to ensure that the eyebrow positions for some combinations were not affected by fatigue?

*Yes, we used two different orders, and also added frequent breaks to avoid fatigue. This information is now added to the Design of the study section.*

7. Have the authors considered the possibility that having an extra person (three as opposed to two) in the room during filming resulted into reduced eyebrow movements for Deaf signers? Or that the hearing signers are more comfortable expressing constructed “anger” emotion due to their experience as interpreters?

*We added a discussion of the first option to the relevant section. The second option seems to be similar to what we already discussed in the original manuscript: the interpreters are more used to the task of performing facial expressions (including emotions) in their professional work.*
