# Supplementary material for: Eyebrow position in grammatical and emotional expressions in Kazakh-Russian Sign Language: A quantitative study
Source: PLoS One. 2020 Jun 2;15(6):e0233731. doi: 10.1371/journal.pone.0233731 (PMC7266324; doi:10.1371/journal.pone.0233731)
Supplement: Peer review history

# Peer review: Decision Letter 0

**DOI:** 10.1371/journal.pone.0233731.r001

**Authors:** Matthew L. Hall

16 Mar 2020

PONE-D-20-02249

Eyebrow position in grammatical and emotional expressions in Kazakh-Russian Sign Language: A quantitative study

PLOS ONE

Dear Dr. Kimmelman,

Thank you for submitting your manuscript to PLOS ONE, and especially for your patience with a review process that was much longer than ideal.  I judged that finding the well-qualified reviewers would ultimately serve this work better than finding less-qualified reviewers who might have had a faster turnaround.  I have now received reviews from three experts in the role of facial expressions in sign languages, and am happy to report that their impression of the manuscript is generally positive: I concur.  Reviewers 1 and 3 request several specific revisions; I encourage you to address them as clearly and specifically as possible in both the revised manuscript and a reply letter.  If your revisions are sufficiently clear, there may not be a need to re-circulate the manuscript for external review.  

We would appreciate receiving your revised manuscript by Apr 25 2020 11:59PM. When you are ready to submit your revision, log on to https://www.editorialmanager.com/pone/ and select the 'Submissions Needing Revision' folder to locate your manuscript file.

If you would like to make changes to your financial disclosure, please include your updated statement in your cover letter.

To enhance the reproducibility of your results, we recommend that if applicable you deposit your laboratory protocols in protocols.io, where a protocol can be assigned its own identifier (DOI) such that it can be cited independently in the future. For instructions see: http://journals.plos.org/plosone/s/submission-guidelines#loc-laboratory-protocols

Please include the following items when submitting your revised manuscript:

- A rebuttal letter that responds to each point raised by the academic editor and reviewer(s). This letter should be uploaded as separate file and labeled 'Response to Reviewers'.
- A marked-up copy of your manuscript that highlights changes made to the original version. This file should be uploaded as separate file and labeled 'Revised Manuscript with Track Changes'.
- An unmarked version of your revised paper without tracked changes. This file should be uploaded as separate file and labeled 'Manuscript'.

Please note while forming your response, if your article is accepted, you may have the opportunity to make the peer review history publicly available. The record will include editor decision letters (with reviews) and your responses to reviewer comments. If eligible, we will contact you to opt in or out.

We look forward to receiving your revised manuscript.

Kind regards,

Matthew L. Hall, PhD

Academic Editor

PLOS ONE

Journal Requirements:

When submitting your revision, we need you to address these additional requirements:

1. Please ensure that your manuscript meets PLOS ONE's style requirements, including those for file naming. The PLOS ONE style templates can be found at http://www.plosone.org/attachments/PLOSOne_formatting_sample_main_body.pdf and http://www.plosone.org/attachments/PLOSOne_formatting_sample_title_authors_affiliations.pdf

2. We note that S1 video includes an image of a participant in the study. 

As per the PLOS ONE policy (http://journals.plos.org/plosone/s/submission-guidelines#loc-human-subjects-research) on papers that include identifying, or potentially identifying, information, the individual(s) or parent(s)/guardian(s) must be informed of the terms of the PLOS open-access (CC-BY) license and provide specific permission for publication of these details under the terms of this license. Please download the Consent Form for Publication in a PLOS Journal (http://journals.plos.org/plosone/s/file?id=8ce6/plos-consent-form-english.pdf). The signed consent form should not be submitted with the manuscript, but should be securely filed in the individual's case notes. Please amend the methods section and ethics statement of the manuscript to explicitly state that the patient/participant has provided consent for publication: “The individual in this manuscript has given written informed consent (as outlined in PLOS consent form) to publish these case details”.

If you are unable to obtain consent from the subject of the photograph, you will need to remove the figure and any other textual identifying information or case descriptions for this individual.

[Note: HTML markup is below. Please do not edit.]

Reviewers' comments:

Reviewer's Responses to Questions

**Comments to the Author**

1. Is the manuscript technically sound, and do the data support the conclusions?

The manuscript must describe a technically sound piece of scientific research with data that supports the conclusions. Experiments must have been conducted rigorously, with appropriate controls, replication, and sample sizes. The conclusions must be drawn appropriately based on the data presented. 

Reviewer #1: Yes

Reviewer #2: Yes

Reviewer #3: Partly

**********

2. Has the statistical analysis been performed appropriately and rigorously? 

Reviewer #1: Yes

Reviewer #2: Yes

Reviewer #3: Yes

**********

3. Have the authors made all data underlying the findings in their manuscript fully available?

The PLOS Data policy requires authors to make all data underlying the findings described in their manuscript fully available without restriction, with rare exception (please refer to the Data Availability Statement in the manuscript PDF file). The data should be provided as part of the manuscript or its supporting information, or deposited to a public repository. For example, in addition to summary statistics, the data points behind means, medians and variance measures should be available. If there are restrictions on publicly sharing data—e.g. participant privacy or use of data from a third party—those must be specified.

Reviewer #1: Yes

Reviewer #2: Yes

Reviewer #3: Yes

**********

4. Is the manuscript presented in an intelligible fashion and written in standard English?

PLOS ONE does not copyedit accepted manuscripts, so the language in submitted articles must be clear, correct, and unambiguous. Any typographical or grammatical errors should be corrected at revision, so please note any specific errors here.

Reviewer #1: Yes

Reviewer #2: Yes

Reviewer #3: Yes

**********

5. Review Comments to the Author

Please use the space provided to explain your answers to the questions above. You may also include additional comments for the author, including concerns about dual publication, research ethics, or publication ethics. (Please upload your review as an attachment if it exceeds 20,000 characters)

Reviewer #1: It is great to see the field for automatic sign language analysis advancing, in particular for the painstaking analyses of facial expressions! My comments below are aimed at polishing this clearly structured and relevant paper.

For ease of reading consider the gender-neutral use of the pronoun “they” as an alternative to s/he.

Lines 77-93 Please note that de Vos et al.’s methodology (specifically sentence types) is not summarized accurately. That is to say, only a subset of the data (polar and content questions) were included in the analysis.

In line 135 the authors claim that “RSL and KRSL are closely related and very similar lexically”. For those unfamiliar with the political history, could you please say something more about when these languages may have started to diverge, and how likely it is that the two communities are still in regular contact.

Line 147-148 Kazakhstan’s government provides 60 free hours of sign language interpreter service support for each deaf person, which could be spent on medical, legal, or other communication needs.

>Are these hours given each year?

Line 200 Did you consider doing a debriefing/post-hoc interview? Why not?

Line 291 What was the frame rate?

Line 357-364 With respect to the pixel analysis, I would like to know whether you have evidence that these are actually perceivable differences for signers themselves?

Line 464-480 Do you think that the interpreters/CODAs may also have reacted differently because they were presented with different stimuli? (written text as opposed to signed videos).

Since you have set it up so nicely, I think it would be worthwhile revisiting de Vos et al.’s Affect over Grammar hypothesis in the conclusion section.

Reviewer #2: This is an excellent addition of a quantitative study of eyebrow movement in the first linguistic study of KRSL which also contributes to the pool of data from several other signed languages to advance the field using more automated means of data gathering then seen in previous studies.

The data were collected with a focus on constructed speech, while most signed language research tried to elicit sentences as if the signed felt the emotion. The authors assert that this allows for emotional expressions that are typical from this particular culture, rather than entering into the debate on whether there are universal facial expressions for emotion.

The methodology is sound for this type of research, and may influence future researchers using computers for data collection and facial measurements.

This was s great glimpse into a signed language that is new to research, and adds more insights into what may or may not change when affect and grammar overlay onto each other in emotional sentence structures. Instead of the affect over grammar suggestions in some prior studies, where grammar disappears, the authors suggest that affect remains within the grammatical structures for polar questions and also statements in KRSL. Wh-questions did not show this differentiation in the average eyebrow movement in sentences, but the authors do acknowledge without a sentence contour analysis this is a matter for future linguistic research. With their frame by frame readings, the data may yet reveal a distinction, as previously found in other signed language research as cited here.

Also, native Deaf signers provided more authentic data than the hearing group, which is not surprising, and in fact is the only data most, if not all, researchers consider.

This is a great addition to the understanding across signed languages, with implications for considering what may be universal and what may be language specific when emotion and grammar interact.

Reviewer #3: The work reported in this manuscript is well motivated, it addresses an important question regarding interaction of affective and linguistic facial expressions during sign language production. Unlike two similar previous studies (De Vos et al. (2009) and Weast (2011), the authors of this manuscript tracked and analyzed subjects’ eyebrow movements during sign production with OpenPose software, which allowed for more precise quantitative and statistical comparisons. Overall, I found this paper to be clearly written. The motivation for the study is well outlined and the results are clearly interpreted.

Only a few minor points:

1. The number of subjects (9) in the study is very small to make some generalizations based on the statistical findings presented in the manuscript; however, the study design and methodology are solid. Given that the authors have explicitly acknowledged some important caveats in their conclusion, the editor may want to consider the manuscript seriously despite a small sample size.

2. Pg 8, Line 328. The number of measurements should be 810. If some measurements were excluded then the authors should state reasons for those exclusions.

3. The authors should include an image in the manuscript (or a video in the supplemental materials) showing how well the OpenPose software tracks the eyebrow movements.

4. For all figures, align the emotion labels to the eyebrows. Some labels were off to one side or another.

5. Did authors analyze left / right asymmetry in the eyebrow positions, particularly for the polar question across the groups? The figure 2 (Hearing signers) seems to show a right-toward asymmetry in the eyebrow position for polar questions, whereas figure 3 (Deaf signers) shows the asymmetry in the opposite direction.

6. Since all signers were instructed to repeat signing a combination of sentence type and emotion 10 times for 9 different combinations. Were those combinations counterbalanced across signers to ensure that the eyebrow positions for some combinations were not affected by fatigue?

7. Have the authors considered the possibility that having an extra person (three as opposed to two) in the room during filming resulted into reduced eyebrow movements for Deaf signers? Or that the hearing signers are more comfortable expressing constructed “anger” emotion due to their experience as interpreters?

**********

6. PLOS authors have the option to publish the peer review history of their article (what does this mean?). If published, this will include your full peer review and any attached files.

If you choose “no”, your identity will remain anonymous but your review may still be made public.

**Do you want your identity to be public for this peer review?** For information about this choice, including consent withdrawal, please see our Privacy Policy.

Reviewer #1: Yes: Connie de Vos

Reviewer #2: Yes: Traci Weast, Ph.D.

Reviewer #3: No

[NOTE: If reviewer comments were submitted as an attachment file, they will be attached to this email and accessible via the submission site. Please log into your account, locate the manuscript record, and check for the action link "View Attachments". If this link does not appear, there are no attachment files to be viewed.]

While revising your submission, please upload your figure files to the Preflight Analysis and Conversion Engine (PACE) digital diagnostic tool, https://pacev2.apexcovantage.com/. PACE helps ensure that figures meet PLOS requirements. To use PACE, you must first register as a user. Registration is free. Then, login and navigate to the UPLOAD tab, where you will find detailed instructions on how to use the tool. If you encounter any issues or have any questions when using PACE, please email us at figures@plos.org. Please note that Supporting Information files do not need this step.

# Author comment: Author response to Decision Letter 0

**DOI:** 10.1371/journal.pone.0233731.r002

11 Apr 2020

We are grateful to the reviewers and the editor for their detailed feedback. Below we address the points raised by the reviewers and describe the changes we implemented. Reviewer 2 did not provide any suggestions for changes, so we do not discuss their review below. All the implemented changes are highlighted with blue text color in the marked up copy of the manuscript. 

Concerning the S1 Video (and also an additional S2 Video, which contains the same material but with OpenPose keypoints overimposed), we have obtained permission from the participant and archived the consent form. The relevant line is added to the Methodology section. 

We have also checked that the manuscript meets the PLOS style requirements. 

Before discussing the reviews, we need to mention a change that we implemented that the reviewers did not ask for. We changed the method of calculating eyebrow height. In the first version, we used the difference of the y-coordinates for the eyebrow keypoints and the top of the nose. This measure could potentially be affected by head and body leans. To address this, we now calculate the distance between pairs of keypoints using both x and y coordinates (the usual distance formula calculation). Note that the results do not substantially change: all the significant effects are in the same direction, but we do find one additional significant interaction, which is also discussed. 

***

Reviewer #1: It is great to see the field for automatic sign language analysis advancing, in particular for the painstaking analyses of facial expressions! My comments below are aimed at polishing this clearly structured and relevant paper.

For ease of reading consider the gender-neutral use of the pronoun “they” as an alternative to s/he.

----Done

Lines 77-93 Please note that de Vos et al.’s methodology (specifically sentence types) is not summarized accurately. That is to say, only a subset of the data (polar and content questions) were included in the analysis.

----Corrected. 

In line 135 the authors claim that “RSL and KRSL are closely related and very similar lexically”. For those unfamiliar with the political history, could you please say something more about when these languages may have started to diverge, and how likely it is that the two communities are still in regular contact.

----We added the basic historical information, but we are not aware of any research discussing these issues. 

Line 147-148 Kazakhstan’s government provides 60 free hours of sign language interpreter service support for each deaf person, which could be spent on medical, legal, or other communication needs.

>Are these hours given each year?

----Yes, the phrase “per year” now added to the sentence. 

Line 200 Did you consider doing a debriefing/post-hoc interview? Why not?

----We did not conduct an extended debriefing because the purpose of the study was disclosed to the participants in advance. The participants did however get the opportunity to ask questions and/or provide feedback. This information is now added in the Design of the study section.

Line 291 What was the frame rate?

----30 fps, information now added to the sentence

Line 357-364 With respect to the pixel analysis, I would like to know whether you have evidence that these are actually perceivable differences for signers themselves?

----The only evidence we have is the validation study described above. We have shown that the signers are able to recognize emotions above the chance level, also in combination with question marking. However, it is impossible to be sure that this recognition is due to perception of eyebrow position alone, because the signers might (and very likely are) using other non-manual clues when recognizing emotions and sentence types. 

Line 464-480 Do you think that the interpreters/CODAs may also have reacted differently because they were presented with different stimuli? (written text as opposed to signed videos).

----This is a possibility, we now added a discussion of it to the relevant section. 

Since you have set it up so nicely, I think it would be worthwhile revisiting de Vos et al.’s Affect over Grammar hypothesis in the conclusion section.

----We have slightly reformulated the discussion of this hypothesis in the Discussion section, and we feel that we do not need to mention it again in the conclusions. 

Reviewer #3: The work reported in this manuscript is well motivated, it addresses an important question regarding interaction of affective and linguistic facial expressions during sign language production. Unlike two similar previous studies (De Vos et al. (2009) and Weast (2011), the authors of this manuscript tracked and analyzed subjects’ eyebrow movements during sign production with OpenPose software, which allowed for more precise quantitative and statistical comparisons. Overall, I found this paper to be clearly written. The motivation for the study is well outlined and the results are clearly interpreted.

Only a few minor points:

1. The number of subjects (9) in the study is very small to make some generalizations based on the statistical findings presented in the manuscript; however, the study design and methodology are solid. Given that the authors have explicitly acknowledged some important caveats in their conclusion, the editor may want to consider the manuscript seriously despite a small sample size.

----We acknowledged the exploratory nature of this research in the manuscript, so it seems that this comment does not require revisions of the manuscript. 

2. Pg 8, Line 328. The number of measurements should be 810. If some measurements were excluded then the authors should state reasons for those exclusions.

----This is correct, the five missing data points are sentences for which OpenPose gave low confidence of detection of keypoints (below 0.7) for all the analyzed eyebrow keypoints in the investigated interval (between 20 and 80% of the video duration). We added this information to the paper in the Statistical Analysis section, and also to the Rmd file with the full description of the analysis. 

3. The authors should include an image in the manuscript (or a video in the supplemental materials) showing how well the OpenPose software tracks the eyebrow movements.

----We included a video in supplemental materials (S2 Video) illustrating OpenPose keypoints overlaid over the same video used in S1 Video. 

4. For all figures, align the emotion labels to the eyebrows. Some labels were off to one side or another.

----We have decided that the figures are more misleading than helpful, and changed them to a more traditional representation of the variables investigated (boxplots). 

5. Did authors analyze left / right asymmetry in the eyebrow positions, particularly for the polar question across the groups? The figure 2 (Hearing signers) seems to show a right-toward asymmetry in the eyebrow position for polar questions, whereas figure 3 (Deaf signers) shows the asymmetry in the opposite direction.

----While we noticed it as well, we had not conducted any analysis prior to manuscript submission. However, we now have conducted a statistical analysis Supplement, and we mention the issue in a footnote in the paper. There is indeed some asymmetry between the eyebrows, and it is influenced by some of the factors considered. We do not however have a good explanation for this finding, so this is left for future research.

6. Since all signers were instructed to repeat signing a combination of sentence type and emotion 10 times for 9 different combinations. Were those combinations counterbalanced across signers to ensure that the eyebrow positions for some combinations were not affected by fatigue?

----Yes, we used two different orders, and also added frequent breaks to avoid fatigue. This information is now added to the Design of the study section. 

7. Have the authors considered the possibility that having an extra person (three as opposed to two) in the room during filming resulted into reduced eyebrow movements for Deaf signers? Or that the hearing signers are more comfortable expressing constructed “anger” emotion due to their experience as interpreters?

----We added a discussion of the first option to the relevant section. The second option seems to be similar to what we already discussed in the original manuscript: the interpreters are more used to the task of performing facial expressions (including emotions) in their professional work.

Attachment Submitted filename: eyebrows PLOS ONE reply to reviews.docx Click here for additional data file.

# Editor report: Decision Letter 1

**DOI:** 10.1371/journal.pone.0233731.r003

**Authors:** Matthew L. Hall

12 May 2020

Eyebrow position in grammatical and emotional expressions in Kazakh-Russian Sign Language: A quantitative study

PONE-D-20-02249R1

Dear Dr. Kimmelman,

We are pleased to inform you that your manuscript has been judged scientifically suitable for publication and will be formally accepted for publication once it complies with all outstanding technical requirements.

Within one week, you will receive an e-mail containing information on the amendments required prior to publication. When all required modifications have been addressed, you will receive a formal acceptance letter and your manuscript will proceed to our production department and be scheduled for publication.

Shortly after the formal acceptance letter is sent, an invoice for payment will follow. To ensure an efficient production and billing process, please log into Editorial Manager at https://www.editorialmanager.com/pone/, click the "Update My Information" link at the top of the page, and update your user information. If you have any billing related questions, please contact our Author Billing department directly at authorbilling@plos.org.

If your institution or institutions have a press office, please notify them about your upcoming paper to enable them to help maximize its impact. If they will be preparing press materials for this manuscript, you must inform our press team as soon as possible and no later than 48 hours after receiving the formal acceptance. Your manuscript will remain under strict press embargo until 2 pm Eastern Time on the date of publication. For more information, please contact onepress@plos.org.

With kind regards,

Matthew L. Hall, PhD

Academic Editor

PLOS ONE

Additional Editor Comments (optional):

Dear Dr. Kimmelman and colleagues - I appreciate your attentiveness to the suggestions for improvement, and the transparency with which you have reported the new analysis. I am very pleased to inform you that after reviewing the changes that you made to the original manuscript in light of the reviewers' comments, I have determined that the manuscript is acceptable for publication, and I thank you for your patience with this process.

Reviewers' comments: The revisions were sufficiently clear that no further review was necessary. 

# Editor report: Acceptance letter

**DOI:** 10.1371/journal.pone.0233731.r004

**Authors:** Matthew L. Hall

20 May 2020

PONE-D-20-02249R1 

Eyebrow position in grammatical and emotional expressions in Kazakh-Russian Sign Language: A quantitative study 

Dear Dr. Kimmelman:

I am pleased to inform you that your manuscript has been deemed suitable for publication in PLOS ONE. Congratulations! Your manuscript is now with our production department. 

If your institution or institutions have a press office, please notify them about your upcoming paper at this point, to enable them to help maximize its impact. If they will be preparing press materials for this manuscript, please inform our press team within the next 48 hours. Your manuscript will remain under strict press embargo until 2 pm Eastern Time on the date of publication. For more information please contact onepress@plos.org.

For any other questions or concerns, please email plosone@plos.org. 

Thank you for submitting your work to PLOS ONE.

With kind regards,

PLOS ONE Editorial Office Staff

on behalf of

Dr. Matthew L. Hall 

Academic Editor

PLOS ONE
